# Developmental Peculiarities and Seed-Borne Endophytes in Quinoa: Omnipresent, Robust Bacilli Contribute to Plant Fitness

**DOI:** 10.3389/fmicb.2016.00002

**Published:** 2016-01-22

**Authors:** Andrea Pitzschke

**Affiliations:** Division of Plant Physiology, Department of Cell Biology, University of SalzburgSalzburg, Austria

**Keywords:** abiotic stress, *Bacillus*, *Chenopodium quinoa* (quinoa), germination, reactive oxygen species (ROS), seed-borne endophyte

## Abstract

Among potential climate change-adapted crops for future agriculture, quinoa (*Chenopodium quinoa*), a facultative halophyte plant with exceptional nutritional properties, stands out as a prime candidate. This work examined how quinoa deals with extreme situations during seed rehydration. Quinoa distinguishes itself from other plants in multiple ways. It germinates within minutes, even under extremely hostile conditions. Broken seeds/split embryos are able to regenerate. Furthermore, quinoa seedlings are resurrection-competent. These peculiarities became in part explainable upon discovery of seed-borne microorganisms. 100% of quinoa seeds, from different sources, are inhabited by diverse members of the genus *Bacillus*. These endophytes are motile and reside in all seedling organs, indicating vertical transmission. Owing to their high catalase activities and superoxide contents the bacteria potentially manipulate the host’s redox status. Superoxide-driven cell expansion enables quinoa to overcome a critical period in development, seedling establishment. Quinoa’s immediate confrontation with “foreign” reactive oxygen species and bacterial elicitors likely induces a naturally primed state, enabling plants to withstand extreme situations. The endophytic bacteria, which are cultivable and highly robust themselves, have high potential for application in agriculture, food (amylase) and cosmetics (catalase) industry. This work also discusses the potential of transferring quinoa’s microbiome to improve stress resistance in other plant species.

## Introduction

### Plant Stress and Seed Germination

The progressive salinization and desertification of land due to climate change will inevitably affect the soil microbiome, soil fauna as well as vegetation. Thus, there is a man-made selection pressure for multi-tolerant extremophiles. Saline and dry soils are poorly accessible for agricultural cultivation. Unlike other environmental factors such as drought, heat, or high light which may influence plant growth at various developmental stages, soil composition, and moisture determines whether seeds give rise to viable plants at all. Drought and high salinity provoke overlapping responses in plants (recently reviewed in e.g., Huang et al., 2012; Golldack et al., 2014). Both stresses are perceived by plant cells as deprivation of water. The lower water potential in saline soil reduces the water availability for the plant. High NaCl concentrations trigger a hyperosmotic shock, which is accompanied by the accumulation of reactive oxygen species (ROS). Similarities also exist in plant adaptation strategies toward high salinity and heavy metal stress (Bose et al., 2014). For this reason halophytes are widely advocated for phytoremediation purposes (Lutts and Lefevre, 2015).

Reactive oxygen species are essential for cellular and long-distance signaling. The role of ROS, including hydrogen peroxide, superoxide and hydroxyl radical, is two-sided (Pitzschke et al., 2006; Bailly et al., 2008). On the one hand, they are critical signaling molecules. On the other hand, ROS at excessive amounts lead to oxidative damage to diverse biomolecules, and ultimately to cell death. To some extent, plants can evade toxic ROS accumulation through ROS-scavenging molecules and enzymes (Mittler, 2002).

Studies in diverse plant species have provided evidence that responses to numerous abiotic and biotic stresses involve ROS production and activation of mitogen-activated protein kinases (MAPKs). MAPKs act both up- and downstream of ROS (Asai and Yoshioka, 2008; Pitzschke and Hirt, 2009), and deficiency or excessive activation is associated with severe developmental defects or abnormal stress phenotypes (Andreasson and Ellis, 2010). Besides mediating stress responses, MAPKs and ROS are also important coordinators of germination. According to a current model, H_2_O_2_ induces a MAPK-dependent decrease in the contents of abscisic acid (a germination-inhibiting hormone). In addition, H_2_O_2_ triggers carbonylation of seed storage proteins, favoring their mobilization (Barba-Espin et al., 2011, 2012). Moderate H_2_O_2_ applications improve germination performance (Barba-Espin et al., 2011), and they can also prevent viability loss in partially desiccated pea seedlings (Roach and Kranner, 2011).

Seed imbibition entails a large increase in ROS contents. For successful germination they must be kept within a certain range that allows ROS signaling. ROS levels above or below the ‘oxidative window for germination’ block development (Bailly et al., 2008). Owing to their ability to react with polyphenols/carbohydrates, ROS also participate in cell wall loosening, which is critical for cell expansion, and thus, plant growth (Tenhaken, 2014).

Furthermore, ROS are essential for priming-mediated resistance, stress acclimation as well as rapid systemic acquired acclimation (Baxter et al., 2014). All these processes rely on the plant’s ability to generate superoxide; accomplished by membrane-bound NADPH oxidases. These enzymes are also crucial for seed germination (Ishibashi et al., 2010; Kranner et al., 2010; Liu et al., 2012a; Singh et al., 2014). Similar membrane-bound enzymes for extracellular superoxide production have recently been discovered in heterotrophic bacteria (Diaz et al., 2013).

### Bacteria in Seed Germination and Plant Development

In their natural habitats, plants are surrounded by myriads of microorganisms that can potentially enter and colonize plant tissues. Most plants become colonized during development. In fact, to be inhabited by microorganisms is rather the rule than the exception and numerous associations between plants and pathogenic or symbiotic fungi or bacteria have been explored (Partida-Martinez and Heil, 2011). The rhizospere represents an important entry point. Soil-borne bacteria, attracted by root exudates, can have a huge impact on plant development. A striking example is the pathogen *Agrobacterium tumefaciens*, which manipulates hormone balances, represses defense mechanisms, and provokes genetic reprogramming in infected host plants; culminating in the growth of tumor-like structures crown-galls (Gelvin, 2012; Pitzschke, 2013). By contrast, rhizobia have growth- and health-promoting effects; they fix nitrogen and mobilize nutrients. Through, biosynthesis of diverse volatile organic compounds microbes cannot only communicate with each, but also with their hosts. Both plant growth- and health-promoting effects are known from certain rhizobacteria (Ryu et al., 2004; Zhang et al., 2007).

Although the role of seed endophytic bacteria still is underestimated, such associations could be beneficial for germination and seedling establishment (Truyens et al., 2015). Seed maturation involves accumulation of starch and a drastic decline in water content. Environmental conditions for plant colonizers thus change substantially, and only endophytes able to withstand high osmotic pressure will be successful seed inhabitants. Characteristic features of some seed-borne bacteria known so far comprise endospore formation, amylase and phytase activity to mobilize starch and phosphorus, respectively, and cell motility to migrate into the seeds before they harden (Johnston-Monje and Raizada, 2011; Truyens et al., 2015). This motility is a main criterion for vertical transmission. In rice, wheatgrass, and maize, respectively, the same bacteria species could be isolated from seeds of consecutive generations (Mukhopadhyay et al., 1996; Johnston-Monje and Raizada, 2011; Liu et al., 2012b; Ringelberg et al., 2012; Gagne-Bourgue et al., 2013).

### Effects of Seed-Borne Bacteria on their Hosts

Despite their beneficial effects on plant health and development and the respective potential for application, seed-borne endophytic bacteria are still largely unexplored. Major effects of seed endophytes on their hosts comprise promotion of growth and protection from stress (reviewed in Truyens et al., 2015). The underlying mechanisms are largely elusive. Growth-promoting effects have been ascribed to seed bacteria from rice (Hardoim et al., 2012), cactus (Puente et al., 2009), and tomato (Xu et al., 2014). In rice, seed endophytes protect young roots from colonization by soil-borne pathogens (Bacilio-Jiménez et al., 2001). Interestingly, besides such antagonistic potential against phytopathogens, seed endophytic bacteria can also alleviate symptoms triggered by abiotic stress. In tobacco, seed endophytes (*Pseudomonas* sp., *Enterobacter* sp.) reduce cadmium phytotoxicity (Mastretta et al., 2009). Long-term cultivation on heavy metal-contaminated soils changes the endophyte composition in seeds of the perennial grass *Agrostis capillaris*. Because selected Cd-resistant seed bacteria (*Bacillus* sp.*, Pantoea* sp.) were found to increase Cd uptake when re-inoculated into Cd-exposed hosts, they hold promise for phytoremediation purposes (Truyens et al., 2014).

Enhanced seed germination arising from seed-borne microorganisms has been related to secretion of bioactive secondary metabolites, or to production of ACC deaminase, which lowers the level of the stress hormone ethylene (*Bacillus*/tomato; Glick, 2014; Xu et al., 2014).

### Quinoa

The pseudo-cereal quinoa is found natively in the Andean region, where it grows at >4000 m above-sea-level. Quinoa is the traditional crop in South America. In the recent past quinoa has witnessed increasing popularity globally, due to its exceptional nutritional properties. The seeds are gluten-free and rich in minerals, proteins, and vitamins (Ruales and Nair, 1992; Vega-Galvez et al., 2010). Quinoa cultivation is spreading worldwide^1^. Being a facultative halophyte, quinoa is able to cope with high levels of salinity and drought stress. Some varieties even accept sea water for irrigation (Adolf et al., 2013). Among potential climate change-adapted crops for future agriculture, quinoa therefore stands out as a prime candidate.

The current work on quinoa germination under non-stress and extreme stress conditions discloses some very unusual plant habits. In the course of experiments, seed-borne endophytes were discovered and their potential contribution to quinoa’s phenotypic peculiarities assessed.

## Materials and Methods

### Plant Material, Growth- and Incubation Conditions

Organic seeds, of the white cultivar ‘Real,’ harvested in Bolivia, were purchased from two independent suppliers (al natura, Austria and Ziegler-Naturprodukte, Germany).

Seeds (app. 20, 10; or single seeds, respectively) were placed into 12-, 24-well or 96-well plates containing sterile imbibition liquid. Alternatively, they were sown onto moist filter paper or YPD agar. Plates were incubated for various periods. All imbibition solutions were freshly prepared before each experiment. Special care was taken to avoid any cross-contamination (single-use equipment). Seeds were considered as germination when their radicles had protruded the seed coat. All experiments, incubations (including bacterial cultivation) and stainings were conducted at 22–24°C.

### Tests of Plant Material for the Presence of Microorganisms

Seed powder was prepared using a mill (Retsch; Germany). Seedlings were surface-sterilized by incubation in 70% ethanol (two times à 5 min, shaking), followed by three washes in distilled water. Seedlings were either macerated (using sterile micro pistils) or cut into segments (with single-use blades) and subsequently incubated on YPD agar.

### Histochemical Detection of Hydrogen Peroxide

Seeds were directly incubated in water containing 1 mg/ml DAB (diluted from a 50-fold stock in water pH 3.8). Alternatively, DAB was added 4 h after seed imbibition in water. Formation of brown precipitates was documented by photography. Experiments were repeated several times (>5).

### Histochemical Detection of Superoxide

Seeds were imbibed in water containing 0.2 mM NBT (NBT itself had no influence on germination). Alternatively, 4-days-old seedlings were incubated in NBT solution. Consistent data were obtained from three independent experiments, and also when using Hepes- or Tris-buffered solution (pH 6.6–7.5) instead of water. Superoxide contents in microbial material were assessed in a similar way, using 0.2 mM NBT solutions.

### Catalase Tests

The formation of bubbles in the presence of exogenous H_2_O_2_ was considered as a (non-quantitative) readout for catalase activity. Seeds were imbibed directly in H_2_O_2_ solution (0.1, 1 mM). For catalase tests with microorganisms, H_2_O_2_ solution was added to colony material collected from seed surfaces or YPD agar. Bubble formation/foaming was documented by photography.

### Microscopy

Microbes proliferating on quinoa seeds were resuspended in water and analyzed using a Leica Linux20 microscope attached to a camera.

### PCR and Taxonomic Classification

DNA was isolated from microorganisms (proliferating on seeds or YPD agar) using a previously published method for genomic DNA extraction from yeast (Lõoke et al., 2011). Alternatively, microbial material was used as a template directly (“colony PCR”). To obtain sequence information from single colonies, dilutions of microbial suspensions were plated on YPD agar. After PCR (conditions: 95°C 5 min, 35 cycles of [95°C 15 s, 50°C 30 s, 70°C 40 s], 70°C 5 min), reaction products were separated on 1x TAE/1% agarose gels and visualized with Midori Green. Primers for DNA sequencing (Microsynth, Switzerland) were 16S_27F (5′-AGAGTTTGATCMTGGCTCAG-3′), or 16S_1492R (5′-RGYTACCTTGTTACGACTT-3′), respectively. Presence of fungal DNA was tested with PCR primers ITS1 (5′-TCCGTAGGTGAACCTGCGG-3′) and ITS4 (5′ TCCTCCGCTTATTGATATGC-3′).

The MultiAlin tool^2^ was used for sequence alignments. Phylograms were subsequently generated using the ClustalW2 tool^3^.

### Detection of Total Protein Profiles

Single colonies, obtained from dilutions of microorganisms proliferating on seeds, were cultivated on YPD agar. Cell material was re-suspended in 1x SDS-loading dye (0.0625 M Tris pH 6.8, 2% SDS, 10% glycerol, 0.1 M DTT, 0.01% bromophenol blue), denatured at 95°C for 5 min, and separated by electrophoresis using 10% SDS-polyacrylamide gels. Gels were subsequently stained with Coomassie Blue R-250. GenBank accession numbers for 16S rDNA sequences are: KU510076-KU510083.

## Results

### Rapid Germination and Early Stress Insensitivity

The initial motivation of this work was to study how an extreme halophyte handles extreme situations at the very onset of its life cycle. To this end, seeds were imbibed in water, or in 200, 300, or 400 mM NaCl. Seed swelling, concurrent with uptake of the surrounding liquids, happened within few minutes. Radicles protruded in the majority of seeds after 30 min; and over the next 2 h roots had clearly elongated (>1 mm). Numerous repeats of the experiment, conducted over a 12-months-period, re-confirmed these observations. Consistently, germination rates were 70–80% both in water and in saline solutions. It was a general rule that seeds showing no radicle emergence within the first 4 h would never germinate. As a comparison, germination was also monitored in amaranth. Quinoa and amaranth share a similar seed architecture (Pal et al., 1990; Prego et al., 1998), and both belong to the Amaranthacae family. Upon imbibition, amaranth seed swelling resembled that of quinoa, both in terms of duration and relative liquid volume absorbed. However, thereafter amaranth could not keep pace with quinoa. In line with previous work by others (Aufhammer et al., 1998), amaranth radicle emergence only became visible after 3 days (**Supplementary Figure S1**).

Quinoa’s germination behavior is not only peculiar in terms of timing, but also in terms of stress sensitivity: NaCl had no adverse effect during this early developmental phase. In fact, NaCl (up to 300 mM) rather seemed to accelerate radicle emergence. However, saline solutions did impair seedling development later on (**Supplementary Figure S2**), suggesting that the treatments *per se* were truly challenging to quinoa. It is a general conception that conditional seed dormancy is a strategy enabling plants to keep the highly sensitive embryo protected until conditions become more favorable. Compared to other developmental stages, plants are very vulnerable during germination. Accordingly, environmental adversities normally block development at the very beginning, i.e., at the germination stage. Likewise, in a given plant species, stress-tolerant ecotypes, transgenics, or mutants frequently exhibit partial insensitivity to the inhibitory effects of stress agents on radicle protrusion AND post-germination development (Carvalho et al., 2010; Duarte et al., 2013; Pitzschke et al., 2014). Quinoa’s unusual germination performance could be applicable to NaCl stress only, or be a more general habit. To test these options, quinoa seeds were challenged with several abiotic stressors at purposely high concentrations that are normally toxic to plants. Surprisingly, imbibition solutions containing 20 mM Pb_2_NO_3_, 20 mM CuSO_4_, or 2 mM CdCl_2_ still enabled radicle emergence and initial axis growth (**Supplementary Figure S3**). In fact, germination even occurred in 10% methanol and isopropanol (not shown), conditions that are certainly inacceptable for plant development and reproduction. What is more, quinoa seeds accepted YPD agar as a growth medium (**Figure 4**; **Supplementary Figures S7** and **S8**). YPD, consisting of yeast extract, peptone and dextrose, represents a rich cocktail of microbial elicitors. Because excessive elicitation of plant immune responses (e.g., ROS production) usually means death, one would consider YPD as highly unsuitable for plants. Accordingly, there was no sign of germination on YPD agar in (viable) seeds of other plant species (amaranth, sesame, millet) over a 14-days observation period.

These observations pointed to a more general and transient “blindness” of quinoa to hostile environments. Though the underlying mechanism remains elusive at this stage, several scenarios can be envisaged: (i) The developmental program is switched on merely “physically,” i.e., by seed swelling and mechanical receptors. Seeds lack sufficient amounts of germination-inhibiting substances; or the embryo is insensitive to such inhibitors. The respective stress signals and receptors are established only *after* radicle protrusion. (ii) Similar to other conditional dormancy-competent plants, quinoa is principally capable of producing and sensing inhibitory compounds. However, these substances are unstable or the respective receptors are temporarily blocked. (iii) Germination may be “enforced” by signals/mechanisms that override any germination-inhibitory program.

### Gas-Producing Activities in Rehydrated Seeds

**FIGURE 1:**
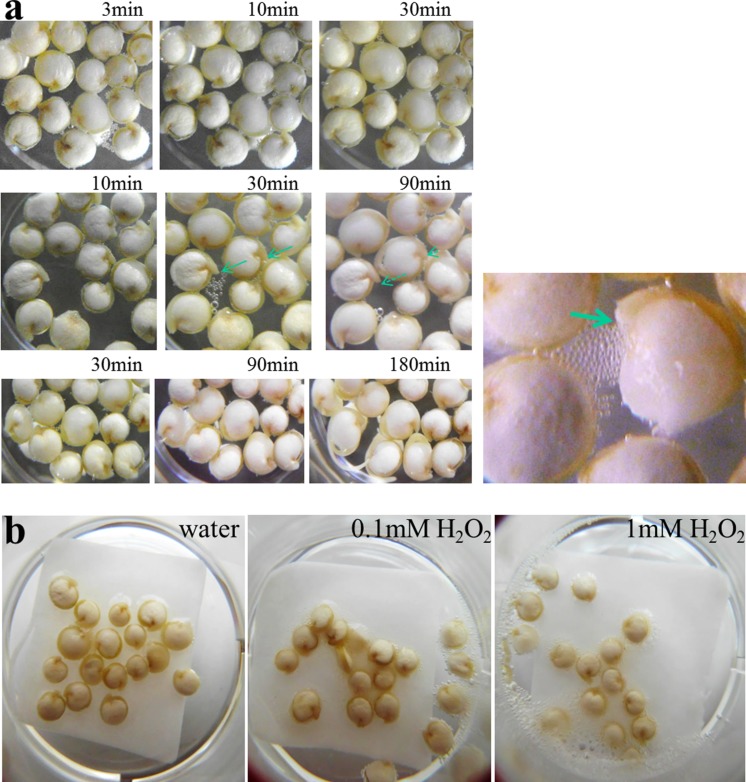
**Early germination and bubble-releasing activities in quinoa.** **(a)** Seeds were placed in water (*t* = 0), and development was monitored in three independent time series experiments as indicated. Representative images are shown. Note seed swelling between the 10- and 30-min time point (middle panel). Radicles during and after the gas-generating phase are indicated by lined and dashed arrows, respectively. Right: close-up view of seedling showing intense air production. The arrow points to the emerging radicle tip. **(b)** Exogenous H_2_O_2_ intensifies air bubble release from seeds. Seeds were imbibed in water, 0.1 mM H_2_O_2_ or 1 mM H_2_O_2_ for 10 min.

**FIGURE 2:**
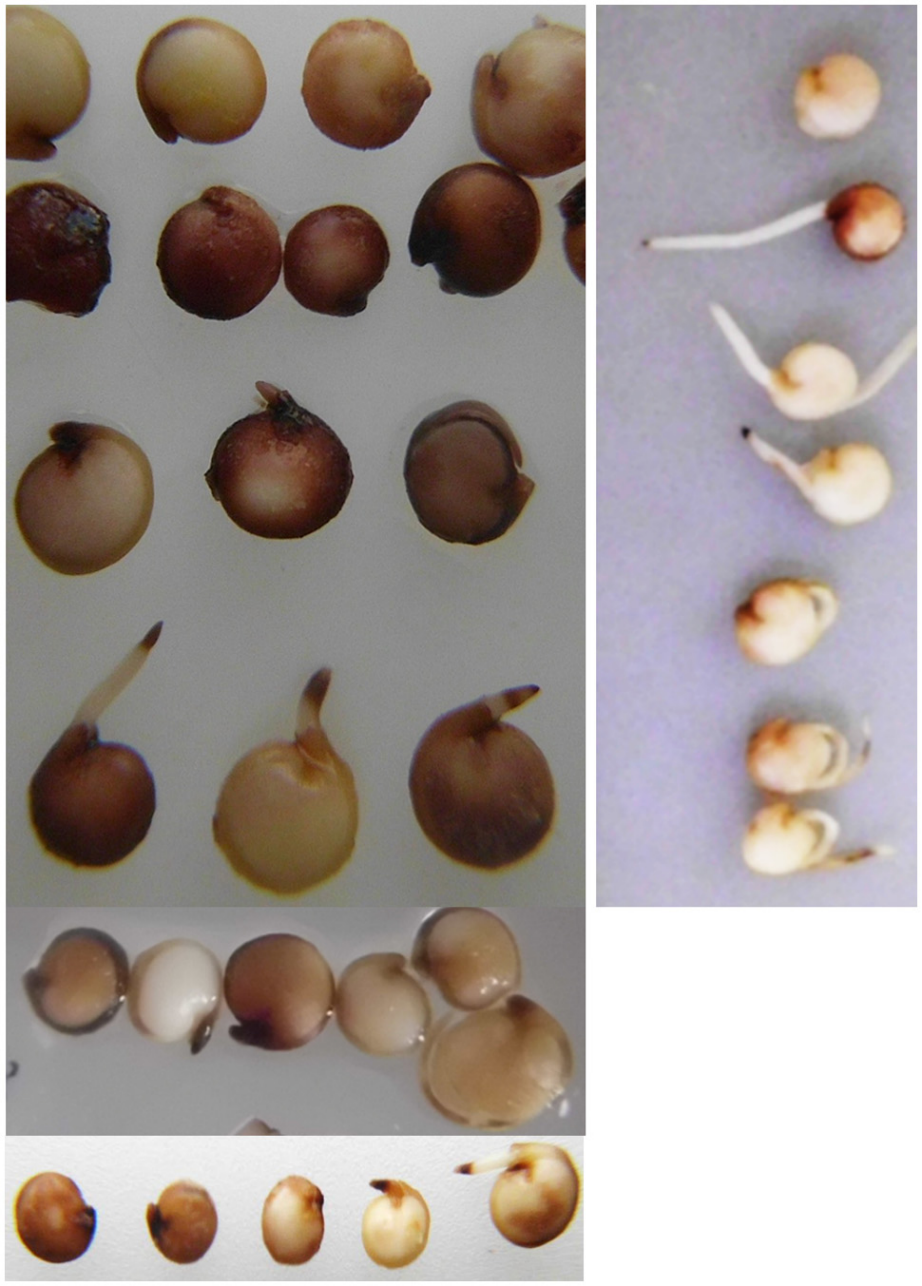
**Histochemical detection of H_2_O_2_ in germinating quinoa.** Seeds were placed for 20 h directly into DAB (1 mg/ml in water) directly **(left)** or after 4 h imbibition in water **(right)**. Brown precipitates indicate the presence of hydrogen peroxide. Some heterogeneity exists in H_2_O_2_ contents and distribution; therefore data of three independent staining experiments are shown.

On the surfaces from imbibed seeds, particularly at the radicle exit site, a progressive release of air bubbles was recognizable. Bubble formation started 1–5 min after seed submergence (in water or saline solution) and ceased after ∼30 min (**Figure 1a**). A simple explanation would be that air, which had been trapped in dry seeds, elapsed through cracks in the seed surface. However, this is very unlikely because bubbles continued to elapse after completion of seed swelling. The volume of gas released from each seed was disproportionately large compared to seed volume, i.e., seeds lack the respective “storage capacity.” Furthermore, killed seeds (30 min 90°C prior to imbibition) generated no bubbles. These observations rather pointed to enzymatic activities; and catalase, which uses H_2_O_2_ to generate water and oxygen, was the suspect enzyme. Its activities are knowingly high in extracts from dry quinoa seeds (Pitzschke et al., 2015), and in-gel activity assays confirmed this (not shown). If “bubbling” indeed resulted from catalase-mediated oxygen formation one would expect a certain pool of the enzyme’s substrate in seeds. Consequently, histochemical stainings were conducted using diaminobenzidine (DAB), which forms brown precipitates in the presence of endogenous H_2_O_2_ and peroxidases. Particularly intensive browning, indicative of local H_2_O_2_ accumulation, appeared on the radicle exit point. Once testa rupture was accomplished, the major site of DAB precipitation shifted to the tip of the growing radicle (**Figure 2**).

If bubbling derived from catalase activity, and if the enzyme’s substrate in seeds was below-saturation levels one would expect exogenous H_2_O_2_ to intensify gas formation. Seeds were therefore soaked in water, or in dilutions of H_2_O_2_. H_2_O_2_-treated seeds showed clearly elevated bubbling activities. The effect was stronger in 1 mM H_2_O_2_ as compared to 0.1 mM H_2_O_2_, indicating that 0.1 mM was still below the enzyme’s saturation limit (**Figure 1b**). Subsequent experiments using a Clarke electrode (oxygen detection) removed any doubt concerning the identity of the arising gas (not shown).

### Resurrection-Competence

**FIGURE 3:**
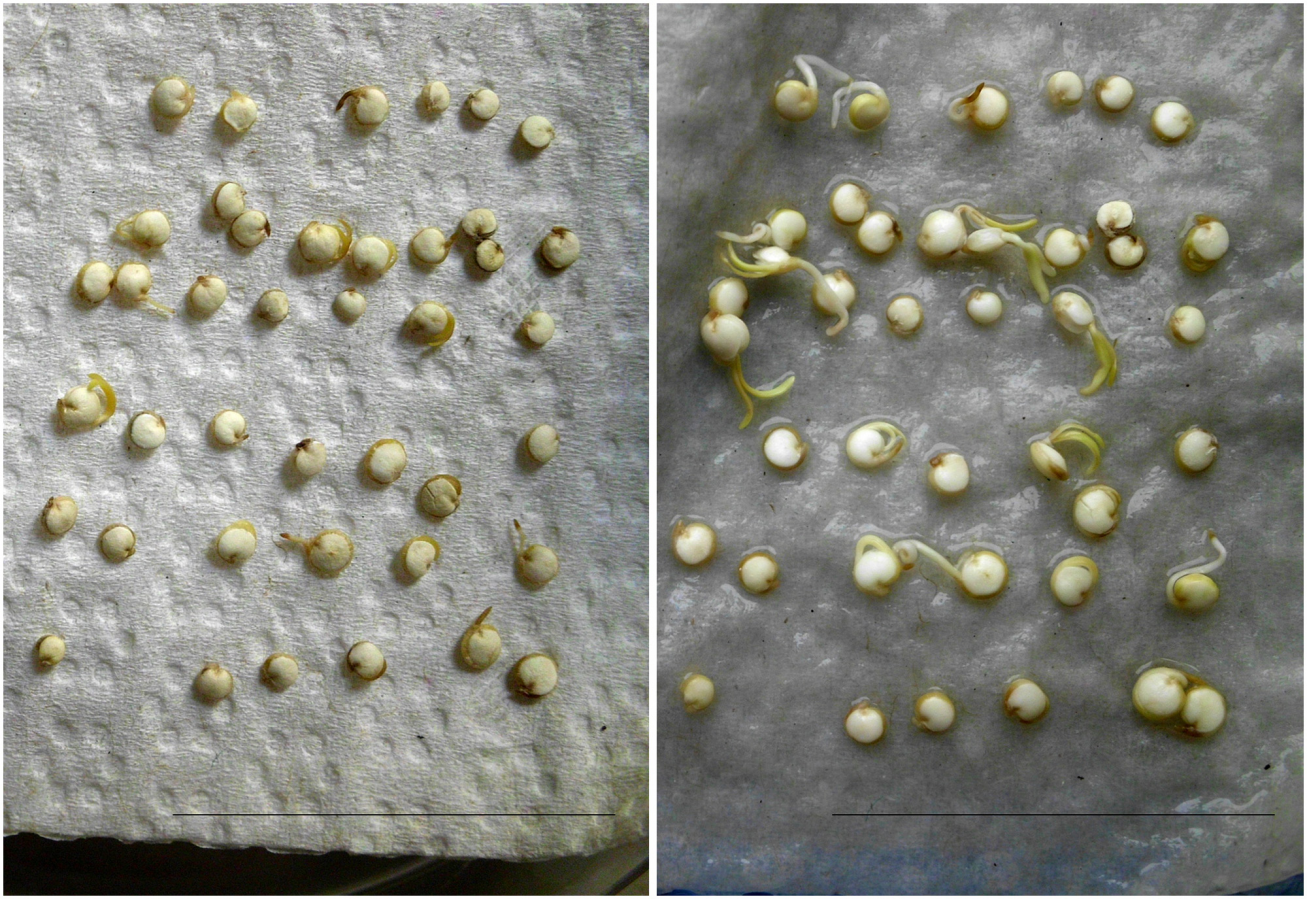
**Quinoa seedlings are resurrection-competent.** Seeds were placed on moist paper tissue to enable germination. After 24 h they had dried-out completely (left). The right photo was taken 20 h after addition of water to fully dehydrated seedlings. Scale bar: 5 cm.

Given the known overlap between plant responses to saline and dry conditions, quinoa seedling performance was examined under extreme drought stress. Seeds were placed on moist paper (to allow germination) and allowed to air-dry completely. Dried seedlings had a crumpled appearance and weighted ∼10% less than completely untreated seeds. Surprisingly, upon re-wetting the fully dehydrated seedlings readily resumed growth (**Figure 3**).

### Regeneration from Broken Seeds – the “Planarian Phenomenon”

**FIGURE 4:**
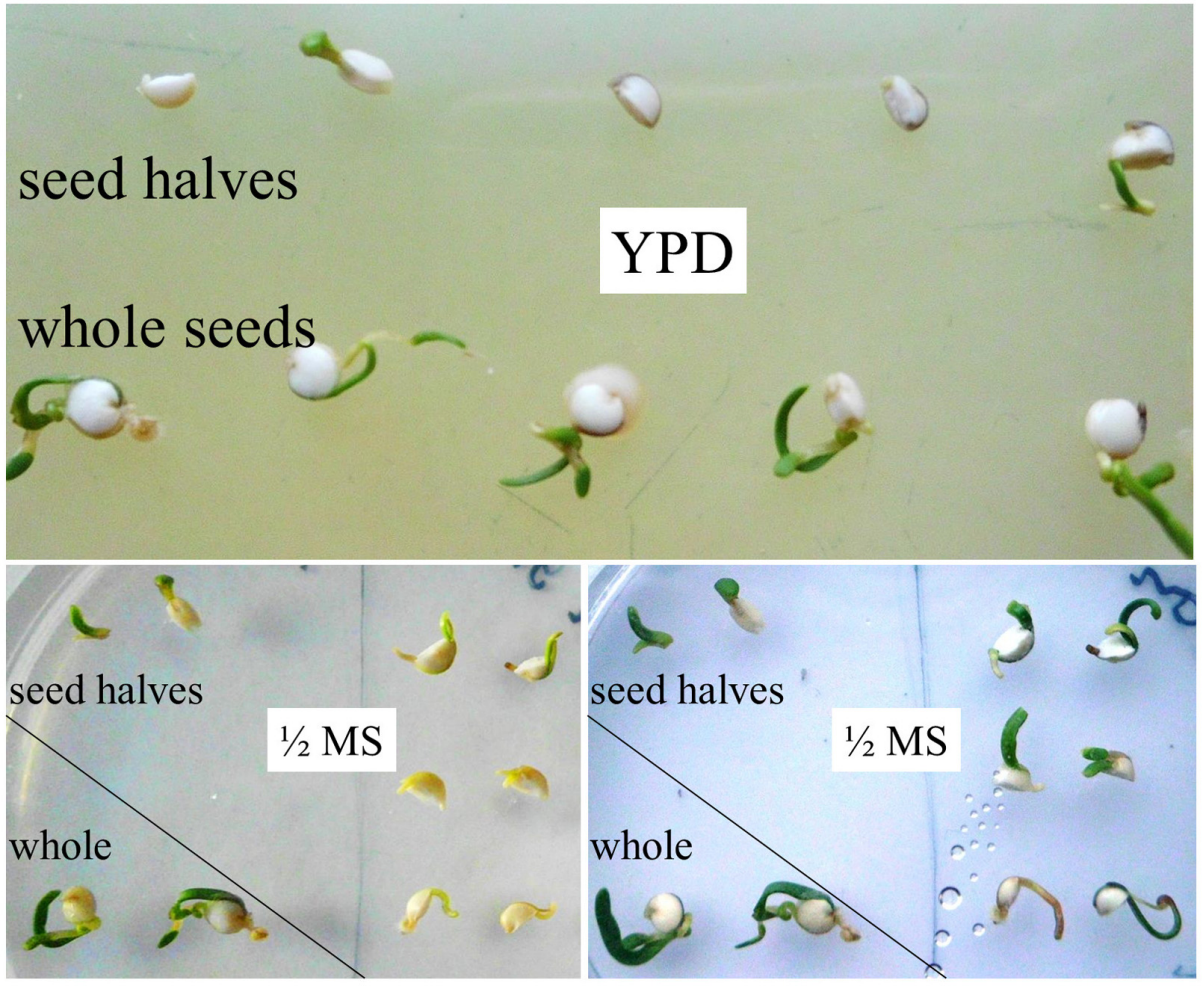
**Regeneration ability of split seeds/embryos.** Intact seeds and seeds split in halves were incubated on YPD agar for 6 days. Instead of releasing/stimulating microbial colonization, seed halves start to grow. Both halves of the same seed have re-generating capacity. Images were taken directly and 20 h after transfer of seed halves from YPD to ½ MS agar (placed pairwise).

Details on quinoa seed architecture have been reported recently (Lopez-Fernandez and Maldonado, 2013). The seed resembles a swollen disk in which the embryo forms a ring embracing the perisperm. Therefore, any vertical cut would not only cleave the perisperm in two. It would also disrupt the embryo and separate essential plant organs. Surprisingly, seed cutting did not abort embryo axes growth in quinoa. Instead, there were leaves developing from both halves of a split seed. They had a healthy green color, indicative of photosynthetic activity. Transfer from YPD to ‘regular’ plant medium (½ MS, 1% agar; **Figure 4**) further facilitated plant growth. To my knowledge, damaged seeds/embryos of no other plant, including Amaranth (same seed architecture) have this capability. For cell culturing and callus propagation plant cells can of course originate from various explants (Moscatiello et al., 2013), but there is no *ad hoc* differentiation from broken seeds. In its ability to re-generate quinoa rather resembles planarians (Reddien and Sanchez Alvarado, 2004; Shomrat and Levin, 2013).

### Seed-Borne Microorganisms

**FIGURE 5:**
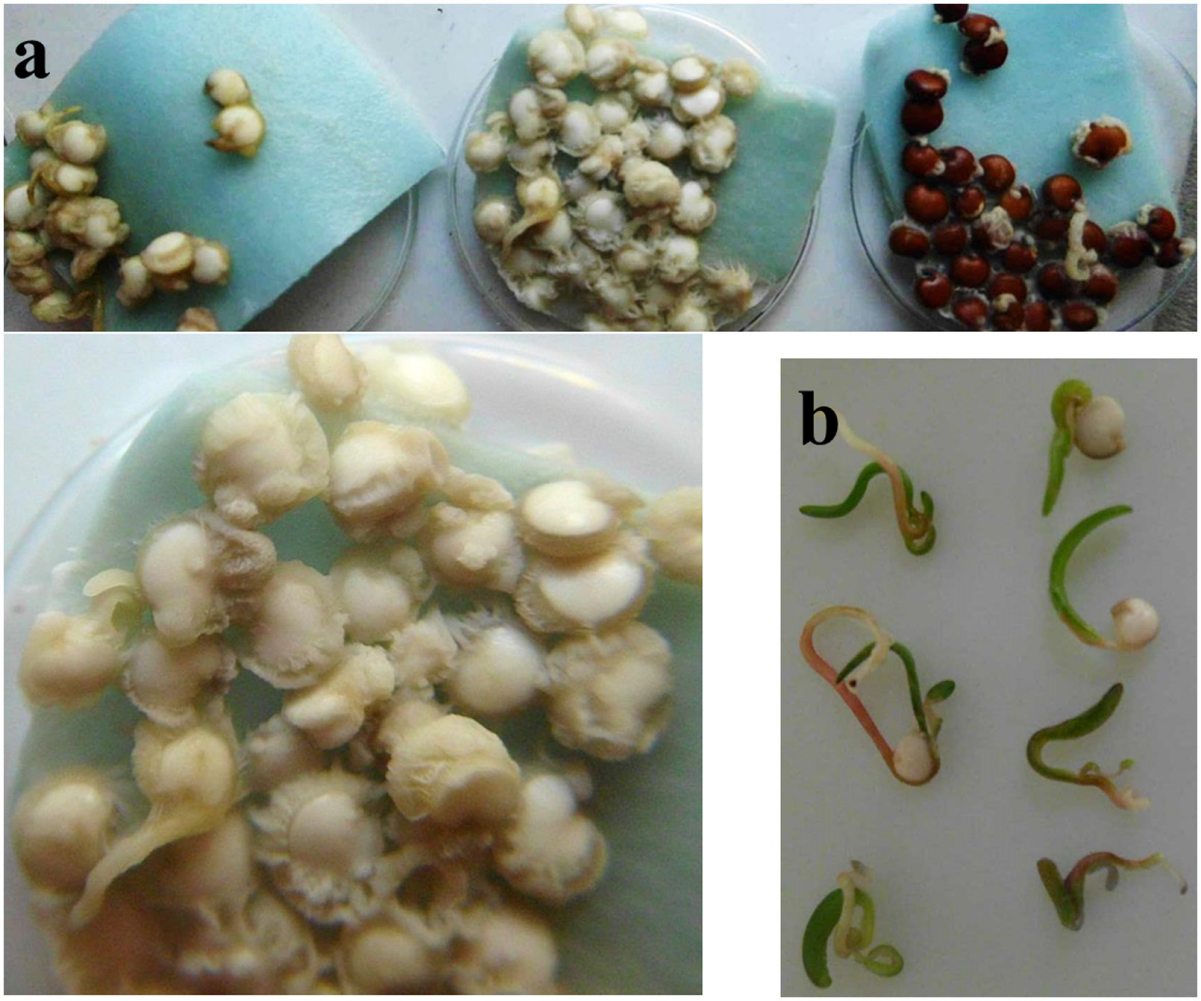
**Humidity-dependent microbial encroachment on quinoa.** **(a)** Seeds were incubated under sterile and high-humidity conditions and photographed after 4 days. White quinoa seeds (left, middle) are from two independent seed batches; one is shown at a higher resolution. **(b)** Seeds grown on agar medium or slightly moistened filter paper develop normally.

Under high humidity conditions (wet filter paper in covered Petri dishes), the previously smooth surfaces of seeds became covered by whitish “bumps.” First appearing after 2 days of incubation these grew appreciably within few hours, indicative of microbial proliferation. At days 3–4, individual seeds became interconnected by a net-like structure (**Figure 5a**). Though still recognizable as such, seed(ling)s fully collapsed upon gentle touch with a tooth pick. With respect to texture and color, seed coat and seed interior became indistinguishable from the soft and whitish microbial mass that was piling up in the surrounding area. Any attempts (e.g., sterile plastics, paper, water from independent sources, change of equipment) to prevent seed colonization under the described incubation conditions failed, a mere “contamination problem” therefore appeared unlikely. More plausibly, the microorganisms originated from the seed interior, as their proliferation was unaffected by seed surface disinfection treatments. Harsh conditions (seed washes in >5% sodium hypochloride for >5 min) proved to be unsuitable, as they blocked germination ability.

Until here experiments had been conducted on pools of 10–30 seeds. In a given sample, microbes released from a single seed can potentially spread and grow on all remaining seeds (“cross-contamination”). To clarify which proportion of seeds actually hosted the microorganisms, seeds were placed one-by-one into separate wells of a 96-well microtiter plate containing sterile water. After 4 days of incubation microbial proliferation was recognizable in each sample. Noteworthy, microbes also emerged from seeds of independent suppliers/batches, as well as on a red quinoa cultivar (**Figure 5a**, right). Their presence was therefore not a seed batch- or storage-related phenomenon, which would have respectively only limited scientific impact. Unfortunately, such constitutive colonization impedes comparative studies between microbe-free vs. microbe-containing quinoa.

To gain insight into this novel plant-microbe association, the microbial partner was characterized in more detail and its potential to account for the observed quinoa growth phenomena was examined.

#### Endophytic Lifestyle and Indications of Movement within the Host

Microbial softening of plant tissue might be indicative of a pathogenic lifestyle. However, this encroachment only occurred under high-humidity conditions (details see Supplementary data file). Seedlings grown in a wet environment failed to reach far enough into the air. Their stems and leaves remained close to the ground, where they became easily “caught” by the towering mass of microbes. A wet environment, in turn, facilitated colony growth. In contrast, low humidity conditions (seeds plated drily on solid plant medium or slightly moistened filter paper) – which better reflect quinoa’s natural habitat – gave rise to normal-looking healthy seedlings (**Figure 5b**).

To assess presence of microorganisms in healthy and symptom-free plants, surface-sterilized individual seedlings were macerated and the resultant liquid examined. Alternatively, root, stem, and cotelydons were separated by cuts with single-use blades (to exclude cross-contamination). With the intention to support growth of any potentially existing microbial organism, plant materials were placed onto YPD agar. In macerated material, colonies appeared after 1 day. After 3 days, colonies also emerged from cut surfaces of seedling segments. They subsequently spread on/around roots, stems as well as cotelydons. This strongly indicates seed-borne microbes to be able to move within the plant. Microbial presence *per se* is harmless to quinoa, as plants lack signs of infection or other developmental impairments. The microbes do not kill their host and *vice versa*, they can be regarded as endophytes. Endophytes are “microbes that colonize living, internal tissues of plants without causing any immediate, overt negative effects” (Bacon et al., 2000).

Knowledge about the existence of an endogenous microbial partner makes quinoa’s peculiarities (rapid germination, early and transient stress insensitivity, bubble release) appear in a new light. Unfortunately, there is no seed material available for studying quinoa in a microbe-free context; and attempts to generate such material (cultivation with antibiotics) have proved unsuccessful. Seed-borne endophytes, by contrast, proliferate *ex*ternally and are cultivable on YPD agar in the absence of plant material. One may thus at least examine their potential contribution to the observed host phenomena.

### Reactive Oxygen Species in Host and Microbes

#### General Consideration

The process of germination involves seed rehydration and emergence of the radicle through the seed coat. It has been shown that embryonic axes growth results from cell elongation rather than cell division (Gimeno-Gilles et al., 2009; Weitbrecht et al., 2011). In part, this is a turgor-driven process, but cell expansion also requires relaxation, i.e., loosening, of the cell walls. Owing to their ability to react with cell wall components, ROS – primarily the short-lived forms, superoxide and hydroxyl radical – play a critical role here. ROS (H_2_O_2_) occurring at moderate levels are used by peroxidases for cross-linking phenolic compounds and glycoproteins causing cell wall stiffening. Stronger oxidative conditions, however, favor formation of superoxide and hydroxyl radical, which directly react with cell wall polymers, causing cell wall loosening (Tenhaken, 2014). Quinoa’s rapid germination and initial plant growth could thus theoretically be attributable to elevated ROS contents. Critical amounts of ROS, in turn, may derive from plant and/or microbial activities. As long they are sufficiently close to the cell wall, these molecules should be equally capable of driving cell expansion, and thus, organ growth. The microbe’s ROS-generating capacity was therefore examined, employing histochemical stainings with nitroblue tretazolium (NBT), which reacts with superoxide to form blue formazane precipitates.

#### Superoxide Contents in Quinoa

**FIGURE 6:**
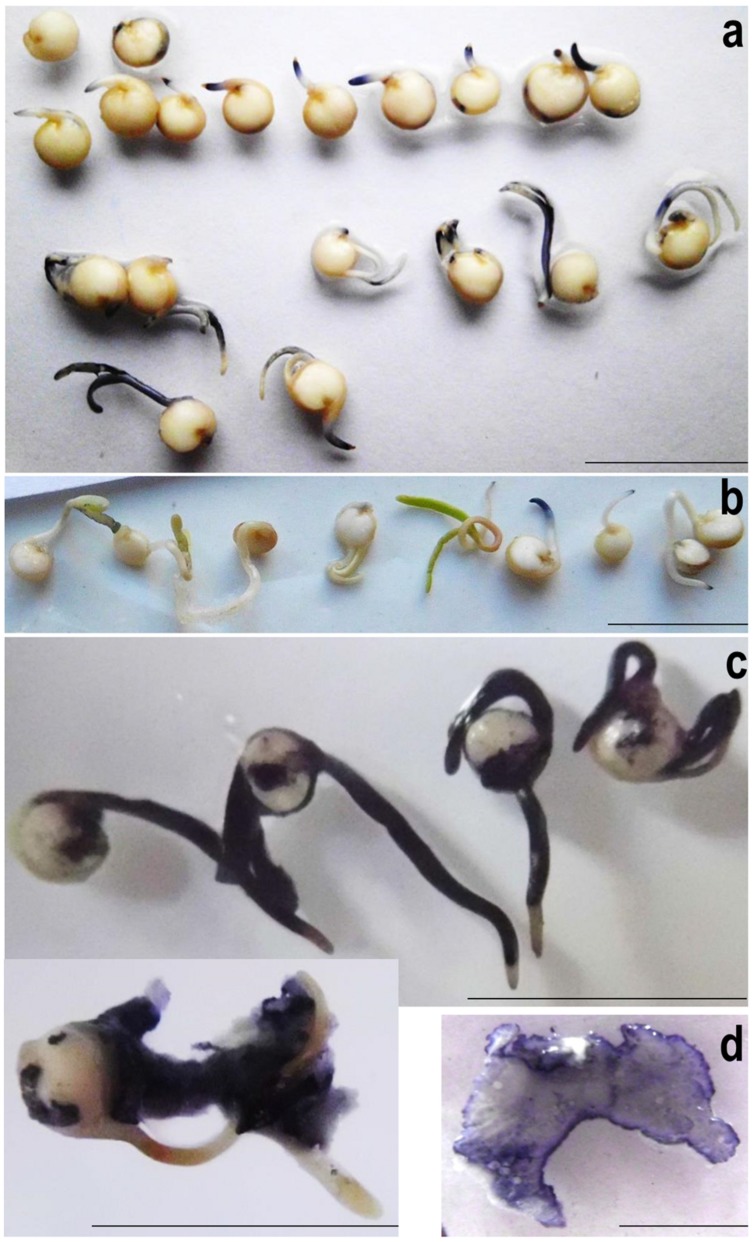
**Superoxide detection in quiona **(a,b)** and seed-borne microorganisms **(c,d)**.** **(a)** Quinoa seeds were imbibed in water containing 0.2 mM NBT for 12 h, arranged according to their developmental stage and photographed. Note the lack of superoxide generation in non-germinating individuals (top; 1 representative shown). **(b)** NBT stainings of 4-days-old seedlings grown on damp filter paper. Experiments were repeated at least three times; one representative is shown. **(c)** Quinoa seedlings heavily colonized by endogenous microbes were incubated in NBT solution for 10 min. The seeds had been germinated on filter paper under high-humidity conditions for three (top) or four (bottom) days. **(d)** Microbial colonies proliferating on YPD agar were stained in NBT solution and photographed after 2 min. Note that color intensity is particularly high at the border, i.e., growing zone. Scale bar: 1 cm.

To monitor superoxide generation during quinoa germination, seeds were directly imbibed in water containing 0.2 mM NBT. Approximately 3 h after imbibition distinct patches of blue appeared. As a comparison, among the fastest accumulators reported so far, *Vigna radiata*, forms visible precipitates only after 10 h of seed imbibition in NBT (Singh et al., 2014). In quinoa, blue color intensity was particularly intense in the distal root zone, suggesting this to be the major site of elongation. Notable amounts of superoxide also occurred in cotelydons, especially when apical axis expansion had contributed to testa rupture (**Figure 6a**). Seeds that failed to germinate produced no color. Superoxide production may therefore be considered as marker for successful germination; and accordingly, NBT stainings as a simple methodology for assessing seed vigor in quinoa. The data corroborate the view that superoxide generation is essential for seed germination and associated growth (Ishibashi et al., 2010; Kranner et al., 2010; Liu et al., 2012a; Singh et al., 2014).

Superoxide was also visualized in healthy 4-days-old quinoa seedlings lacking obvious signs of colonization. Upon seedlings submergence in NBT solution some patches of blue had formed after 1–2 h (**Figure 6b**). Contrasting the situation in imbibed seeds (see above), color intensity remained weak also after prolonged incubation, and there were no distinct zones of blue. Thus, despite hosting microbial cells and the respective elicitors, quinoa seedlings apparently maintain a “normal” redox balance. Superoxide accumulation is most pronounced during cell expansion-driven growth, i.e., germination and the early development.

#### Microbes have High Superoxide Levels

Next, superoxide contents were investigated in quinoa’s microbial partner, using heavily colonized seed(ling)s (grown on wet filter paper or YPD). Upon addition of NBT solution, samples turned blue within seconds, reaching maximum color intensity (dark-blue) after 5 min (**Figure 6c**). In their function as cell wall-loosening agents, ROS released from seed-borne microbes may thus assist radicle protrusion and cell expansion. Under conditions unfavorable to plant development, microbial ROS likely facilitate tissue softening, thus making nutrients available for proliferation. Considering that superoxide and its even more aggressive side-product OH^.^ are destructive to DNA, proteins and other biomolecules, its accumulation seen here, even under non-challenging conditions, appears unusually high. However, *extra*cellular ROS generation – and respective limited intracellular damage – had recently been discovered in bacteria (Diaz et al., 2013).

Although quinoa endophytes are strong ROS-generators and knowingly present inside symptom-free plants (see above), superoxide levels were low in 4-days-old seedlings (**Figure 6b**). As a likely explanation, healthy plants carry few microbial cells only. It should also be pointed out that microbial superoxide production is strongest during proliferation (**Figure 6d**). Inside living host tissues the microbes likely proliferate much less “aggressively,” as compared to outside.

### Microbial Catalase Activities as Likely Cause of Bubble Release from Seeds

Catalases are wide-spread among living organism (Klotz et al., 1997). Against this background I examined a possible involvement of endophytes in “seed bubbling”. When freshly grown colony material was immersed in H_2_O_2_ solution, bubble formation started immediately. The foam that built up within seconds points to strong microbial catalase activities. Observations were largely identical with colonies proliferating on seeds or YPD medium (**Supplementary Figure S4**).

### Quinoa Endophytes belong to the Genus *Bacillus*

The final question was which type(s) of microorganisms inhabited quinoa seeds and thus could account for the developmental phenotypes observed. Despite strong similarities in colony morphology to some yeast strains growth (Honigberg, 2011) (**Supplementary Figure S5**), microscopy and PCR-based analyses revealed exclusive presence of bacterial populations. To get an idea on population diversity, I used DNA-homology-based classification and protein profiling analyses. Microscopy of microbial populations revealed certain differences with respect to cell shape (roundish vs. stretched) and motility (slow, rapid, or extremely rapid). Cells had the tendency to aggregate, and cell sizes (600–700 nm width) spoke for bacterial rather than fungal origin (**Supplementary Figure S6**). To nevertheless assess the possibility of co-existing fungi among the bacterial cells, PCR experiments were conducted using standard primers (fungITS1/ITS4) for the amplification of fungal ribosomal RNA genes. Reactions were set up in parallel with primers for bacterial ribosomal RNA (bact16S_27f/1492r).

**FIGURE 7:**
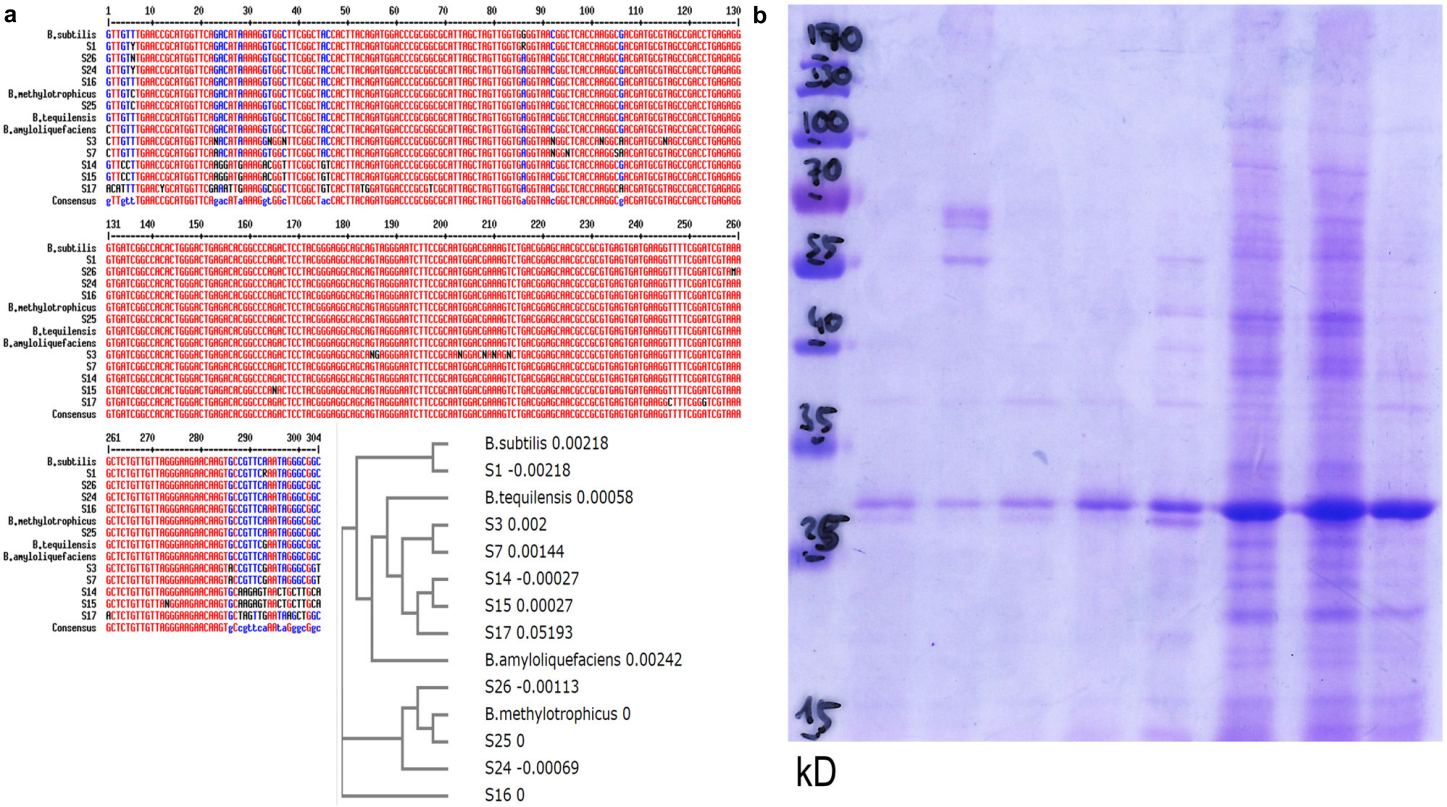
***Bacillus* population diversity in quinoa seeds.** **(a)** 16S rDNA sequences (hypervariable region) from bacteria proliferating on quinoa seeds, or subcultured single colonies (S14–S17, S24, S25, S26). Alignment and phylogram include representatives of the genus *Bacillus*: *B. amyloliquefaciens* sensu strico TM7 AY055225, *B. subtilis* gi| 61744006, *B. methylotrophicus* gi| 916445157, *B. tequilensis* gi| 385866544. See also sequence deposits in GenBank and **Supplementary Table S1**. **(b)** Protein profiles of randomly selected bacterial isolates.

Template material originated from colonies that had been proliferating on imbibed seeds. More precisely, these were two independent microbial communities for each of two seed batches. Consistent with results from microscopy, fungal DNA-directed primers yielded no detectable product. Bacterial 16S rDNA-targeting primers generated products of the expected size (1.5 kb) in all four templates. Based on their 16S rDNA sequence homology quinoa endophytes belong to the genus *Bacillus.* Notably, heterogeneity existed within and between individual DNA samples, even though microbial template material originated from the same seed batch or even the same seed. This suggested co-existence of multiple bacterial isolates. Consequently, single colonies (obtained via diluting and sub-culturing) were used for 16S rDNA sequence analysis, homology search, and alignments (**Figure 7a**; **Supplementary Table S1**, GenBank submissions). All samples turned out to represent the genus *Bacillus*, and closest homologs were *Bacillus subtilis, B. amyloliquefaciens*, *B. methylotrophicus*, or *B. tequilensis*.

To further explore microbial population diversity, I used comparative protein profile analysis. Such approach had proven useful in a similar context in maize (Figueiredo et al., 2009). In their protein patterns, individual colonies originating from a single seed (**Figure 7b**) clearly differed from each other. One may interpret this pattern heterogeneity as a first indication of strain-specific sub-tasks. Notably, all colonies examined (>50) had high superoxide contents. They also consistently showed catalase activity; consistent with the homology-based classification as *bacilli* (Jedrzejas and Huang, 2003).

It is evident that the here-presented collection of sequences and protein profiles is not exhaustive. Quinoa’s microbiome in its entirety can only be elucidated using high-throughput techniques (e.g., NGS). The current data suggest co-existence of several microorganisms in quinoa, most/all of which are *bacilli*.

More precise statements on the identity of quinoa seed-borne microbes cannot be made at this stage. As highlighted previously, there are “difficulties of purely 16S rDNA-based taxonomy, emphasizing the need to interpret the massive amounts of molecular data from environmental sequencing projects in a bacterial ecology framework” (Maughan and Van der Auwera, 2011). Future work will therefore employ other marker genes for a more detailed taxonomic classification; accompanied by further biochemical characterization.

## Discussion

Quinoa demonstrates in multiple ways that our knowledge on plant habits is anything but complete. Most of us would presumably have considered some phenomena as “very odd” or “simply impossible.” Quinoa distinguishes itself from other plants in terms of germination time (rapid) and flexibility (hostile/artificial environments tolerated), seedling growth (fast) and bubble release during early imbibition. In addition, broken seeds/split embryos are able to regenerate, and seedlings can revive from an air-dry state. The latter ability knowingly exists only in a small heterogeneous group of so-called resurrection plants (Dinakar and Bartels, 2013). Quinoa would thus be the first crop plant with resurrection-competence. Ongoing research – of obvious practical relevance – shall determine until which developmental stage quinoa holds its ability to revive.

Endophytic bacteria belonging to the genus *Bacillus* were found to reside in 100% of seeds, from different batches and cultivars. It is tempting to speculate that the association is obligate for the host. Because these bacilli are mobile and omnipresent their vertical transmission is very likely. It remains to be seen whether (seed-originating) microbes can enter plants anew, i.e., via roots. This would enable them to make their way into previously “naïve” plants and resultant progenies.

The fact that under low/moderate humidity bacteria stay within the plant may explain why they remained unnoticed so far. Under conditions found in quinoa’s natural habitat (the Andes; arid climate), both plant and microbe may benefit from this endophytic association. Bacilli can persist as spores over a long time (Gest and Mandelstam, 1987). Seeds appear as an ideal means for storage but also for distribution. Cells exiting from germinating seedlings are potential starting material for colonization of a (new) soil environment. To pioneer new grounds the endophyte must permit host survival (seed production).

Under high-humidity conditions quinoa seedlings performed rather poorly. The microbial partners made the best of the situation by exploring its host as a mere energy source. Seed coat softening owing to bacterial activities enabled them to access nutrients also from outside. Soon, plant material became covered under layers of microbes. This seems an efficient for microbial proliferation, but it is not a sustainable strategy because it means death of the host before seed production. How long the bacilli can survive and form spores outside their host remains elusive. Cells are cultivable on YPD (**Supplementary Figure S5**), but in nature (soil) nutrients are rather scarce. What is more, quinoa’s natural habitat is hostile, not only for plants. If microbes want to survive and proliferate, they must withstand the same demanding environment as their host. At least for high-salinity stress this is indeed the case.

**FIGURE 8:**
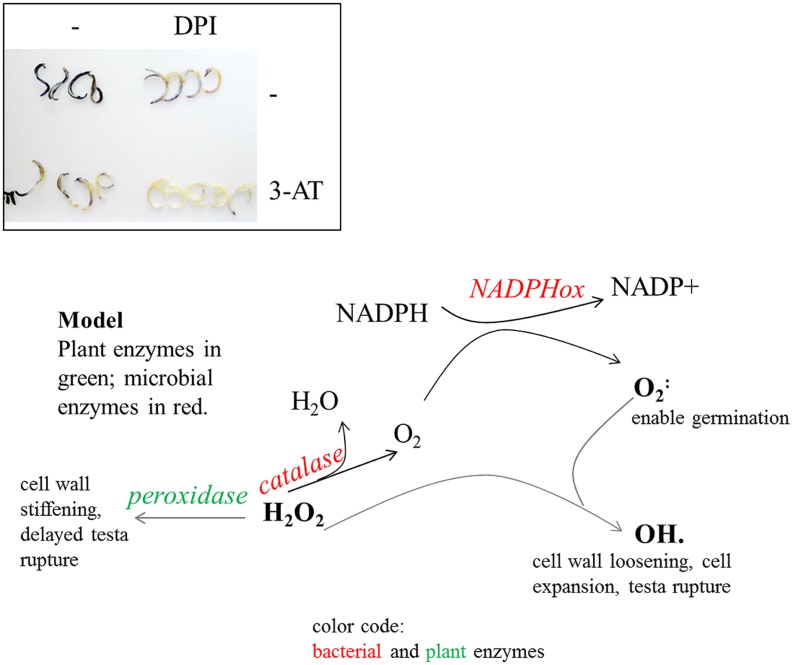
**Inhibitor studies and a derived model on ROS-regulatory activities in quinoa and seed-borne endophytes.** Top: superoxide detection after inhibitor treatments. Seeds were imbibed for 20 h in water, 0.5 mM DPI [NAD(P)H oxidase inhibitor], and/or 10 mM 3-AT (catalase inhibitor). Embryos were subsequently released and stained for 2 h in NBT solution. Superoxide levels are diminished in DPI- as well as in 3-AT-treated seeds; they are barely detectable when both inhibitors are co-applied. These data suggest NAD(P)H oxidase activities (of plant and/or bacterial origin) to be the main source of superoxide accumulation during germination. The seed interior has only limited access to oxygen. Bacterial catalase may serve to replenish the oxygen pool and to turn the seed interior aerobic. H_2_O_2_ levels exceeding enzymatic conversion react with superoxide to form hydroxy radicals (OH^⋅^), which facilitate cell growth. Bacterial catalases shift H_2_O_2_ conversion toward ROS production and cell expansion rather than peroxidase-mediated detoxification and cell wall stiffening. Being aerobic organisms they rely on oxygen which is abundantly available only AFTER testa rupture. Bacteria also contribute to H_2_O_2_ removal, thereby limiting exposure of cells to potentially toxic amounts of ROS.

The general impression was that quinoa is “forced” to grow, irrespective of its environment. Though final proof is lacking (see below), a model can be proposed based on the current data: owing to rapid cell elongation, stimulated by bacterial superoxide, quinoa overcomes the most sensitive stage in plant development within short time (**Figure 8**, model). It is a general conception that plants withstand stresses better when encountered later in development. Its exceptional resurrection-competence makes quinoa even more flexible. It remains to be seen whether quinoa endophytes have additional, i.e., redox-independent mechanisms, to manipulate host growth. Notably, members of the genus *Bacillus* are known to produce plant growth-promoting substances (Chen et al., 2007; Madhaiyan et al., 2010).

The endophytic association *may* protect quinoa from other – potentially pathogenic – microorganisms, thereby ensuring seed viability and plant health. This presumption derives from the known ability of plant endophytes to produce antimicrobial substances (Mousa and Raizada, 2015) and the virtual absence of other microorganisms in/outside quinoa seeds. Irrespective of seed surface sterilization, fungi or obviously different bacteria emerged only rarely (<1%), even when seeds were incubated on sugar-containing medium, YPD- or LB-agar. Resuspended seed powder gave no rise to any colony growth (>14 days incubation on YPD or LB).

A very instructive review article compiling reports on bacterial seed endophytes made clear that to harbor a single bacterial strain is rather the exception. Among the 25 host plant species known so far, 19 are inhabited by multiple endophytes, including Actinobacteria, Bacteroidetes, Firmicutes, and Proteobacteria. Proteobacteria (particularly gamma-) are the most predominant ones isolated form a wide variety of plants. Quinoa adds an economically important crop to the list of host plants. Its endophytic population exhibits important features for seed colonizers (reviewed in Truyens et al., 2015). These include amylase activity to utilize starch and resume growth after long-term survival in dry seeds, cell mobility to migrate within the plant and to enter seeds before seed hardening, and the ability to withstand high osmotic pressure arising from accumulation of starch and water loss during seed maturation.

Indications for Quinoa endophyte amylase activities are their ability to fully soften quinoa seeds, whose major part, the centrally located perisperm, consists of starch-enriched dead cells (Lopez-Fernandez and Maldonado, 2013) as well as sequence homology to *B. amyloliquefaciens*. Furthermore, quinoa endophytes accept starch as a sole energy source (AP, unpublished).

Cell motility is high. (In fact it was barely possible to get a sharp microscopy image). Being able to form spores (as observed by microscopy), quinoa-derived bacteria are well-prepared for long-term survival and osmotic pressure. They also exhibit high stress resistance in their vegetative stage, as concluded from the rapid encroachment on seeds imbibed in high-salinity solutions. In addition, quinoa endophytes withstand other potentially toxic adversities, as they also proliferated on CdCl_2_-treated seeds. Because quinoa itself displays high tolerance toward heavy metal stress the potential of this novel plant-endophyte-association for phytoremediation deserves further attention.

It will be interesting to see whether *all* quinoa plants growing worldwide harbor seed endophytes, and whether the microbiome in highly salt-tolerant quinoa cultivars differs from that in less tolerant cultivars. Such specificity would indicate that plants owe their robustness to an ingenious combination of bacteria. An attractive hypothesis arising from these considerations is: are bacterial strains from highly tolerant quinoa cultivars transferable and effective, i.e., tolerance-enhancing, in less resistant cultivars?

An intriguing question concerns the microbe’s capability to enter species other than quinoa. Not necessarily would other hosts benefit from such association. To stay healthy, the host must be able to actively maintain its redox balance (i.e., flexibly adjust its own ROS producing and scavenging activities) and/or tolerate major fluctuations in ROS concentrations. In other words, quinoa and its endophyte may be a perfect match, but the same endophyte in a different plant might be a disaster. Ongoing research therefore involves cocultivation of several plant species with quinoa endophytes, assessment of disease symptoms and stress performances (AP, unpublished). It remains to be seen whether the bacteria can establish themselves in these plants. As highlighted very recently, inoculation of microbiomes into gnotobiotic hosts holds huge potential to improve plant health (Mueller and Sachs, 2015). A major bottleneck for such approach, cultivability, would not be a hurdle for quinoa endophytes.

As microbial cells are present in seeds already, they can potentially active the plant’s immune system from the very beginning on. Flagellin and other bacterial elicitors, undoubtedly present also on the surface of quinoa endophytes, can be perceived by respective MAMP (microbe-associated molecular patterns) receptors of the host cells. It is tempting to speculate that signaling pathways, e.g., those involving MAPKs (Pitzschke et al., 2009), are activated to turn the plant into a state of alert. In other words, endophyte-hosting seeds are ‘naturally’ primed. As a result, plants arising from such seeds may tolerate stress better as compared to non-inhabited seeds. Quinoa endophytes do not just ‘reside’ in plant tissue, but move within the plant and are metabolically active. Time-dependent changes in MAMP identity and composition are therefore likely to occur; and new MAMPs can activate other host receptors. In the long run, numerous stress signaling pathways should become activated and mediate host resistance to multiple stresses. In this context it is worth mentioning a recent review (Wiesel et al., 2014), which highlights the potential of biological elicitors for crop protection. One reason for the high salinity tolerance in quinoa plants is their low stomatal density (Shabala et al., 2012). Because (at least in *Arabidopsis*) elicitor-inducible MAPK proteins have a second function in stomatal pattern control (Wang et al., 2007; Pitzschke, 2015), an indirect contribution of quinoa to limit water loss appears possible.

Leading scientist in the field believe that ‘quinoa has some unique as yet unidentified features’ which account for its outstanding salt tolerance (Adolf et al., 2013). In the current work several findings and considerations suggest endophytic partners to be one of these features, and to account for the observed phenotypes. For an ultimate proof one would have to cure quinoa from its endophytes and then use naïve seeds for re-inoculation. This is not trivial because harsh sterilization procedures prevent seed germination, antibiotics severely affect seedling growth, and plants develop poorly in autoclaved soil.

## Author Contributions

AP discovered quinoa endophytes, designed and conducted all experiments and wrote the paper. Helpful comments/assistance by others are listed under “acknowledgments.”

## Conflict of Interest Statement

The author declares that the research was conducted in the absence of any commercial or financial relationships that could be construed as a potential conflict of interest.

## Footnotes

1 http://www.fao.org/quinoa/en/

2 http://multalin.toulouse.inra.fr/

3 http://www.ebi.ac.uk/
