# Supplementary material for: Developmental Peculiarities and Seed-Borne Endophytes in Quinoa: Omnipresent, Robust Bacilli Contribute to Plant Fitness
Source: Front Microbiol. 2016 Jan 22;7:2. doi: 10.3389/fmicb.2016.00002 (PMC4722091; doi:10.3389/fmicb.2016.00002)
Supplement: Supplementary file 1 [file Data_Sheet_1.PDF]

Supplemental data file (text, figures, table)

High humidity favours bacterial encroachment

Under conditions (such as high humidity), where plant growth cannot keep pace with microbial proliferation, quinoa is seemingly forced to surrender. Comparative test were therefore conducted with dry and pre-imbibed seeds (20 hours, water) on YPD agar. The reasoning was that pre-imbibition should provide a humid environment to seed-borne microorganism, and YPD facilitate their proliferation. Concurrent with quinoa germination, microbial proliferation proceeded on/around pre-imbibed plant material. Colonies emerged from each of >50 seeds tested, re-confirming their universal occurrence in quinoa. Similar to observations with wet filter paper (figure4 a), microbes emerging from seeds on YPD agar spread rapidly (suppl. figure S7), piled up and softened the thereby enclosed seedling (suppl. figure S7b). Despite their muddy texture, such seedlings retained a healthy look; their leaves stayed green throughout. Furthermore, leaves only turned soft when heavily covered by the microbial net. Plant organs reaching above this net retained their rigid texture (suppl. figure S7c), suggesting that microbes staying inside the plant cause no damage while excessive external colonization is destructive. The associated cut from air supply presumably aggravates the situation.

Contrasting rapid encroachment on pre-imbibed seeds, there was barely endogenous proliferation on drily-placed seeds. Six days after sowing on YPD agar “clean” seedlings had developed from the majority of seeds (90/100; from 4 experiments á 25 seeds). For the remaining ones it is valid: Once colonies occurred, they spread similarly fast as those exiting from pre-imbibed seeds.

That absence of colony growth from drily-plated seeds could be attributable to a complete microbial entrapment within the seed coat is highly unlikely. First, since seeds successfully germinate, seed-borne microbes would have the site of radicle protrusion as a possible exit point. Second, seeds that had been split in halves remained non-colonized (figure 5). With the intention to facilitate microbial exit from dry seeds and microbial contact with growth medium, seeds were applied in powder form onto YPD agar. Over the entire six-day-observation period there were no colonies emerging. Assuming humidity to be critical for colony formation, seed powder was first re-suspended in water, incubated for 0, 7, or 20 hours, and subsequently dotted (2µl) on YPD agar. None of these samples gave rise to colony

growth. It is therefore tempting to speculate that microbial proliferation needs to be initiated by host signals which are active only in growing, living tissue. The question how long quinoa takes to generate such signals was subsequently addressed in time series experiments involving seed imbibition for different duration (1 up to 6 hours), followed by incubation on YPD agar. Colony emergence, first detectable after 24 hour, was found to be independent of the imbibition period. Irrespective of whether seeds had been pre-imbibed for 1 or 6 hours, they became heavily colonized after 42 hours on YPD (suppl. figure S8). One may therefore conclude that host signals inducing microbial proliferation are produced in sufficient amounts within one hour.

Quinoa endophyte sequence	% identity	Gene ID;	Homolog name	Isolation source
**S1**	99% (1207/1220 bp)	gb|KT719890.1|	Bacillus methylotrophicus strain MSL_3065	Spacecraft surface
**S2**	934/955(98%)	gb|KR258764.1|	Bacillus sp.LW-14 16S	Coal mine
**S3**	688/714(96%)	gb|KU182821.1|	Bacillus sp. LMR315	Heavy metal multi-polluted soil
**S7**	1156/1160(99%)	gb|KT720350.1|	Bacillus tequilensis strain V48	Spacecraft collection
**S14**	1019/1021(99%)	gb|JX860235.1|	Bacillus sp. LAMA 762	Atlantic marine bacteria
**S15**	706/709(99%)	gb|KT720198.1|	Bacillus aerophilus strain M1.12b	Spacecraft collection
**S16**	1116/1119(99%)	gb|KT719451.1|	Bacillus methylotrophicus strain MER_TA_47.1	Spacecraft collection
**S17**	1113/1128(99%)	gb|KT216027.1|	Bacillus sp. LB15	midgut
**S24**	1056/1059(99%)	gb|CP007244.1|	Bacillus amyloliquefaciens subsp. plantarum TrigoCor1448	Plants (Brazil)
**S25**	1054/1063(99%)	gb|KU146560.1|	Bacillus methylotrophicus strain R1B 16S	Endophyte from *Dendrobium huoshanense*
**S26**	1113/1128(99%)	gb|KT216027.1|	Bacillus sp. LB15	Endophyte from *Eupatorium odoratum*

Suppl. table S1

Closest homologs of 16S ribosomal RNA gene sequences of quinoa-derived bacteria.

Legends of supplementary figures

Suppl. figure S1

Comparison of germination time and stress sensitivity between Amaranth and Quinoa

Seeds were incubated on filter paper soaked with water or 200 mM NaCl for the indicated periods.

Suppl. figure S2

Abiotic stress inhibits seedling development

Seeds were directly sown on moist filter paper, soaked with water, 200mM NaCl or 200µM $CdCl_2$. The photo was taken at day 5.

Suppl. figure S3

Quinoa germination under extreme situations

Seeds were placed on filter paper soaked with the indicated solutions and photographed after 5 hours and 6 days. (The 400mM NaCl, 4d sample contains a contamination).

Suppl. figure S4

Microbes have catalase activity.

The bacterial ´volcano´ (see figure 5b) ten seconds after addition of H_2O_2 solution

Suppl. figure S5

Quinoa endophytes have sophisticated colony morphology and are cultivable

left: Microbes exiting from quinoa seeds on YPD agar. The photo was taken at the 6th day of incubation. Scale bar: 1cm

right: Re-streaked colonies are able to grow independently on YPD agar.

Suppl. figure S6

Microscopy image of quinoa endophytes

Imbibition solutions of microbially colonized seeds (four days after rehydration in water and incubation under high-humidity conditions). Scale bar: 10µm

Suppl. figure S7

Growth of quinoa on YPD and progressive colonization by seed-borne endophytes.

a) Seeds were imbibed in water for 24, transferred to YPD agar and photographed after 24h and 48h.

b) While drily-sown seeds (left) remain non-colonized, microorganisms rapidly proliferate on pre-imbibed seeds to form volcano-like structures (top- and side-view).

c) Seed(ling)s covered under massively proliferating colonies turn soft.

d) Moderately colonized seeds (from YPD agar, microbes wiped off prior to photography) whose leaves had reached into the air retained a rigid tissue texture.

Suppl. figure S8

Pre-imbibition favors proliferation of seed-borne microbes.

Quinoa seeds were placed on YPD agar, directly or after imbibition in water for 1 to 6 hours. The plate was photographed after 24h, 30h and 42h. Magnification/arrow: first colonies detectable at the seed surface of one seed after 24h, on several seeds after 28h.

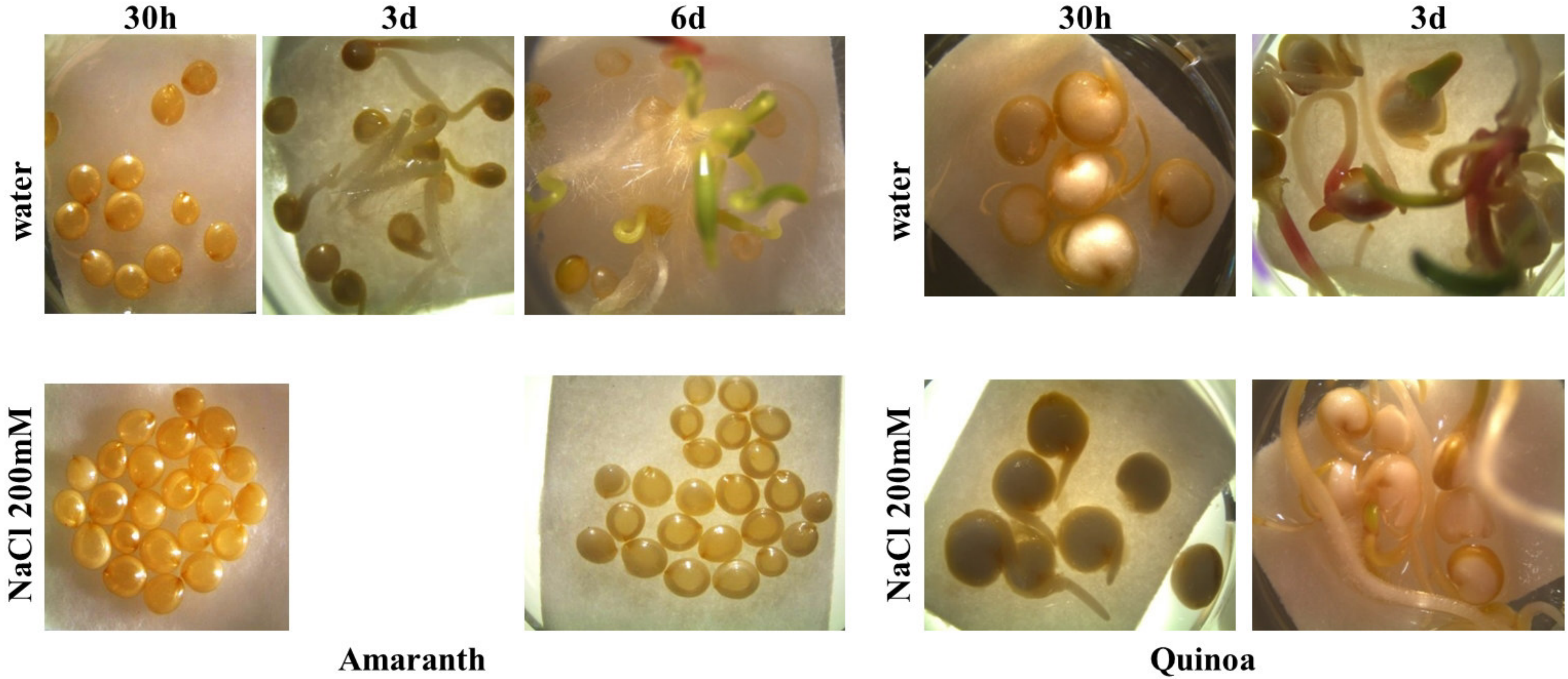

Suppl. figure S1

Comparison of germination time and stress sensitivity between Amaranth and Quinoa

Seeds were incubated on filter paper soaked with water or 200 mM NaCl for the indicated periods.

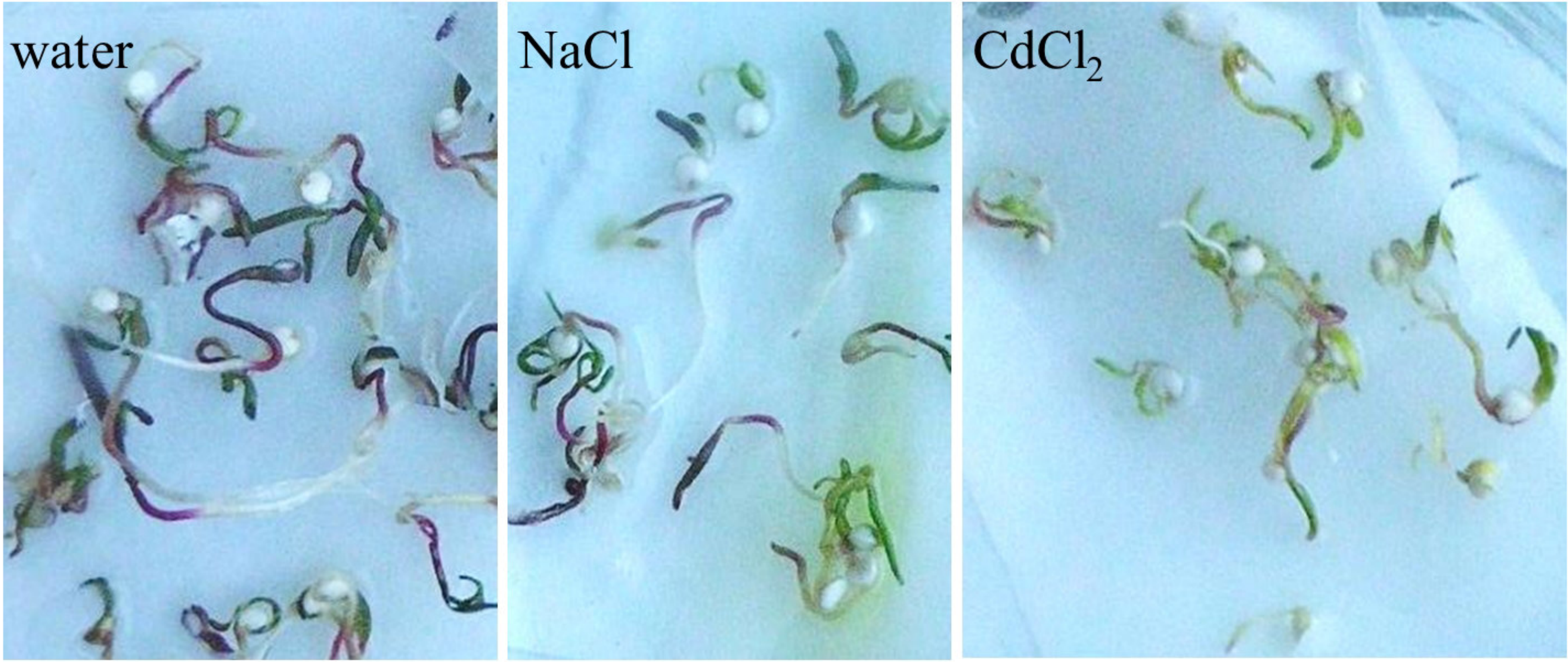

Suppl. figure S2

Abiotic stress inhibits seedling development.

Seeds were directly sown on moist filter paper, soaked with water, 200mM NaCl or 200µM $CdCl_2$. The photo was taken at day 5.

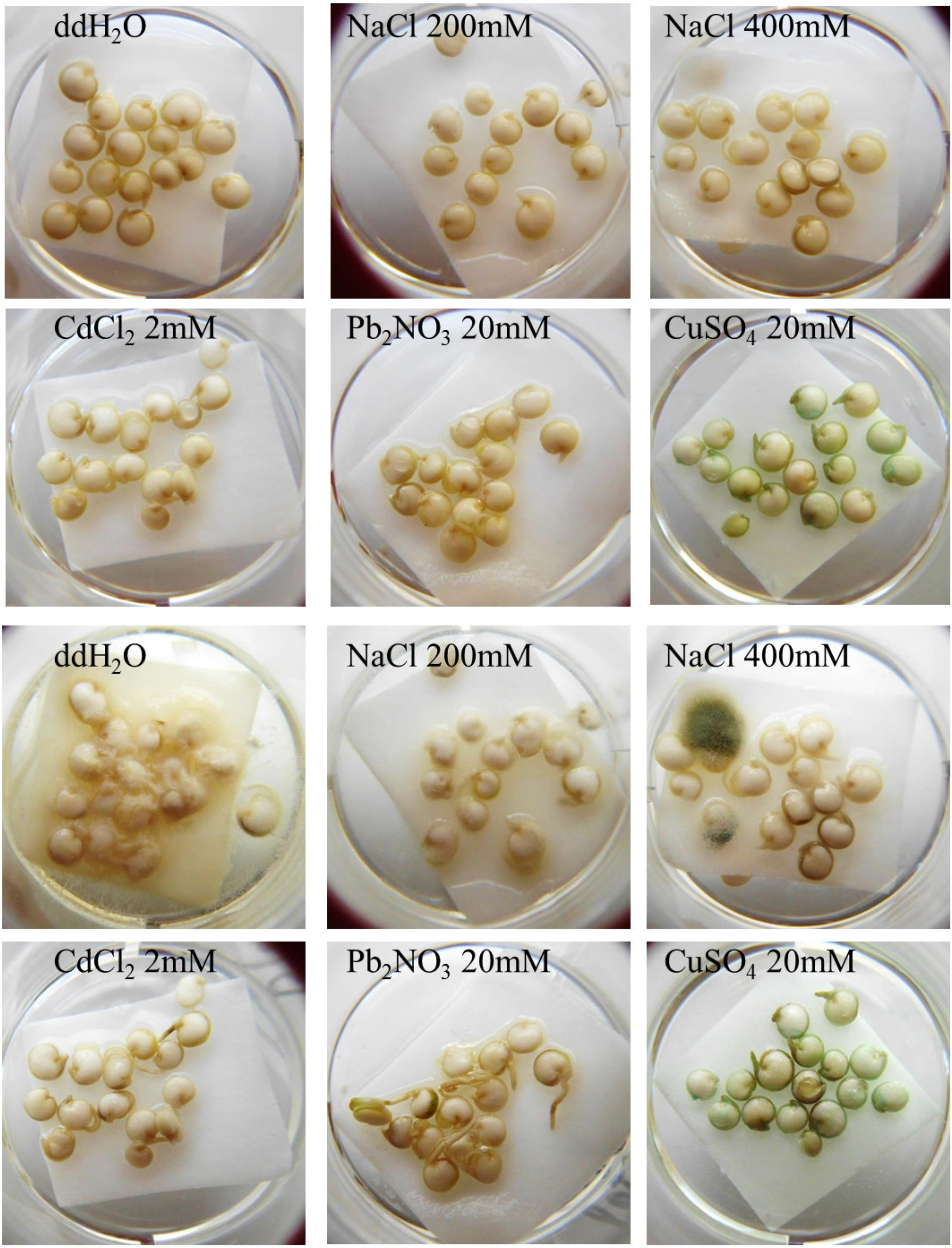

Suppl. figure S3

Quinoa germination under extreme situations

Seeds were placed on filter paper soaked with the indicated solutions and photographed after 5 hours and 6 days. (The 400mM NaCl, 4d sample contains a contamination).

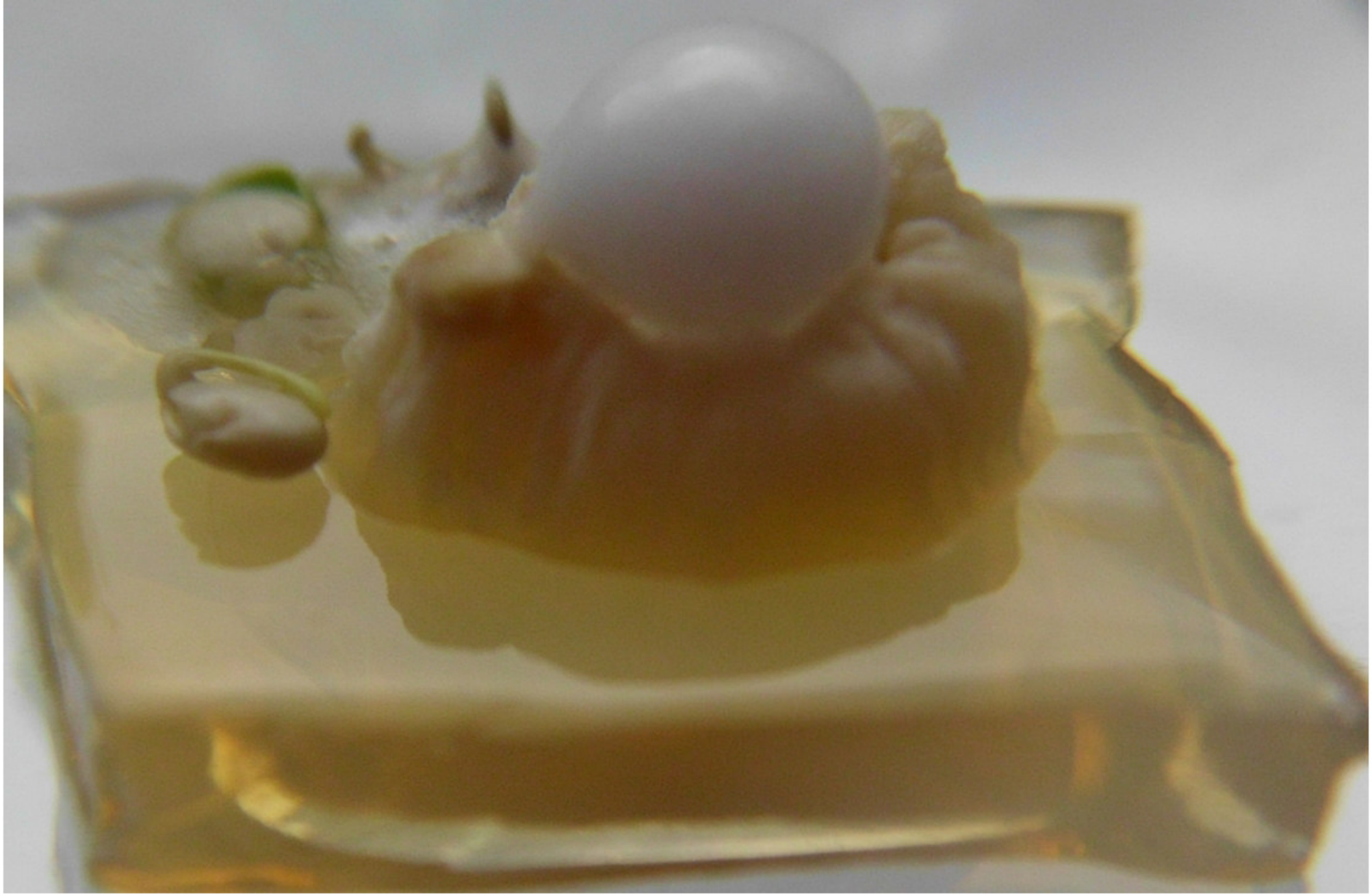

Suppl. figure S4
Microbes have catalase activity.
The bacterial ´volcano´ (see figure 5b) ten seconds after addition of H_2O_2 solution.

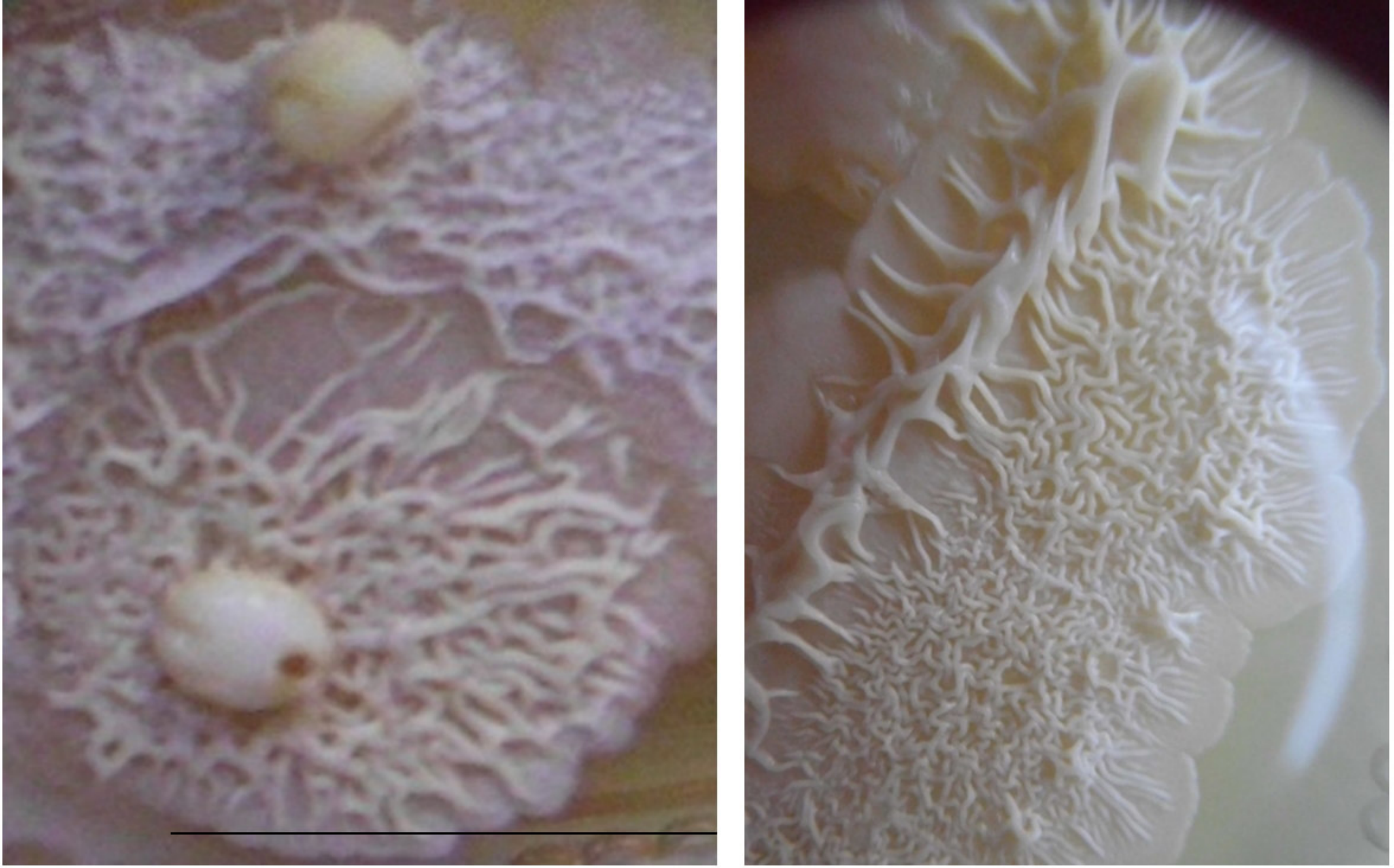

Suppl. figure S5

Quinoa endophytes have a sophisticated colony morphology and are cultivable.

left: Microbes exiting from quinoa seeds on YPD agar. The photo was taken at the 6th day of incubation. Scale bar: 1cm

right: Re-streaked colonies are able to grow independently on YPD agar.

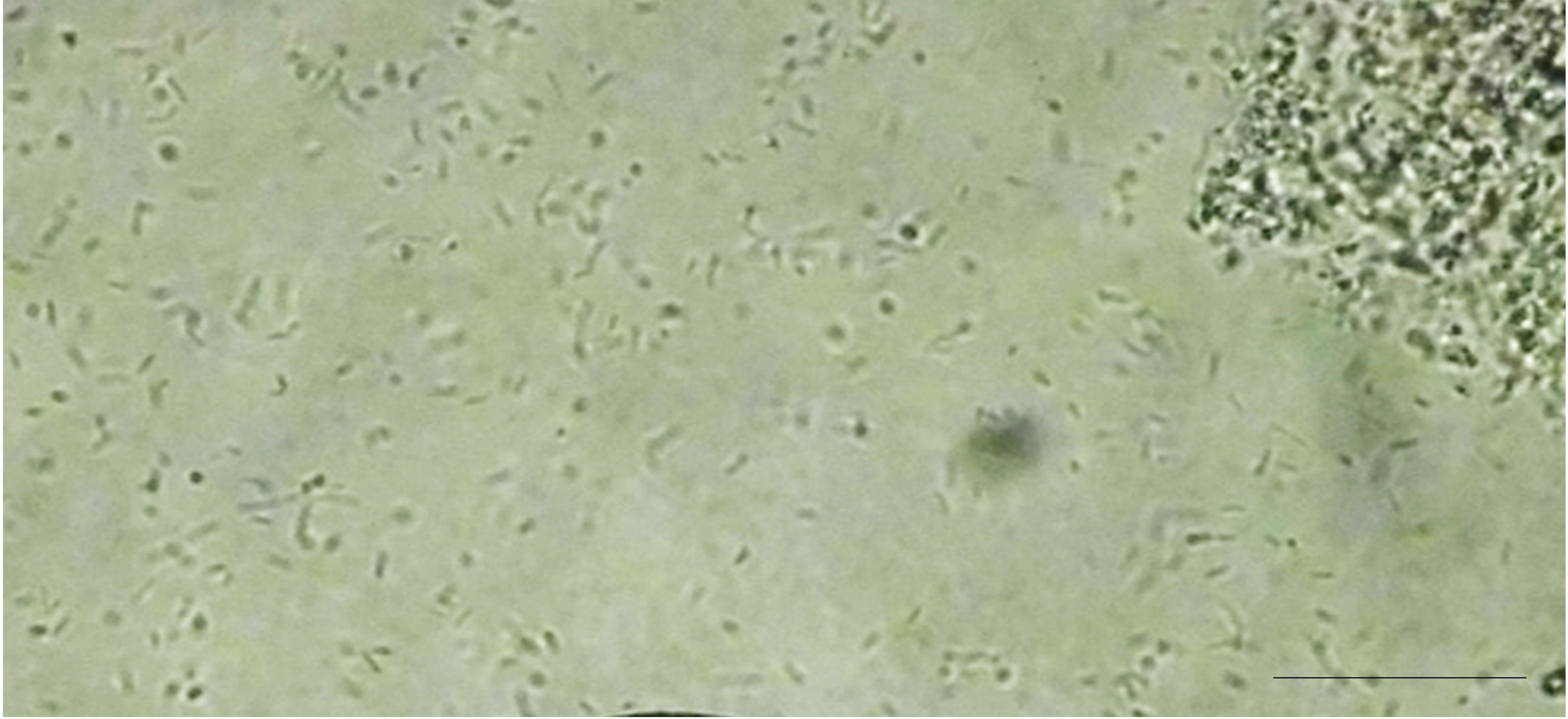

Suppl. figure S6
Microscopy image of quinoa endophytes
Imbibition solutions of microbially colonized seeds (four days after rehydration in water and incubation under high-humidity conditions). Scale bar: 10µm

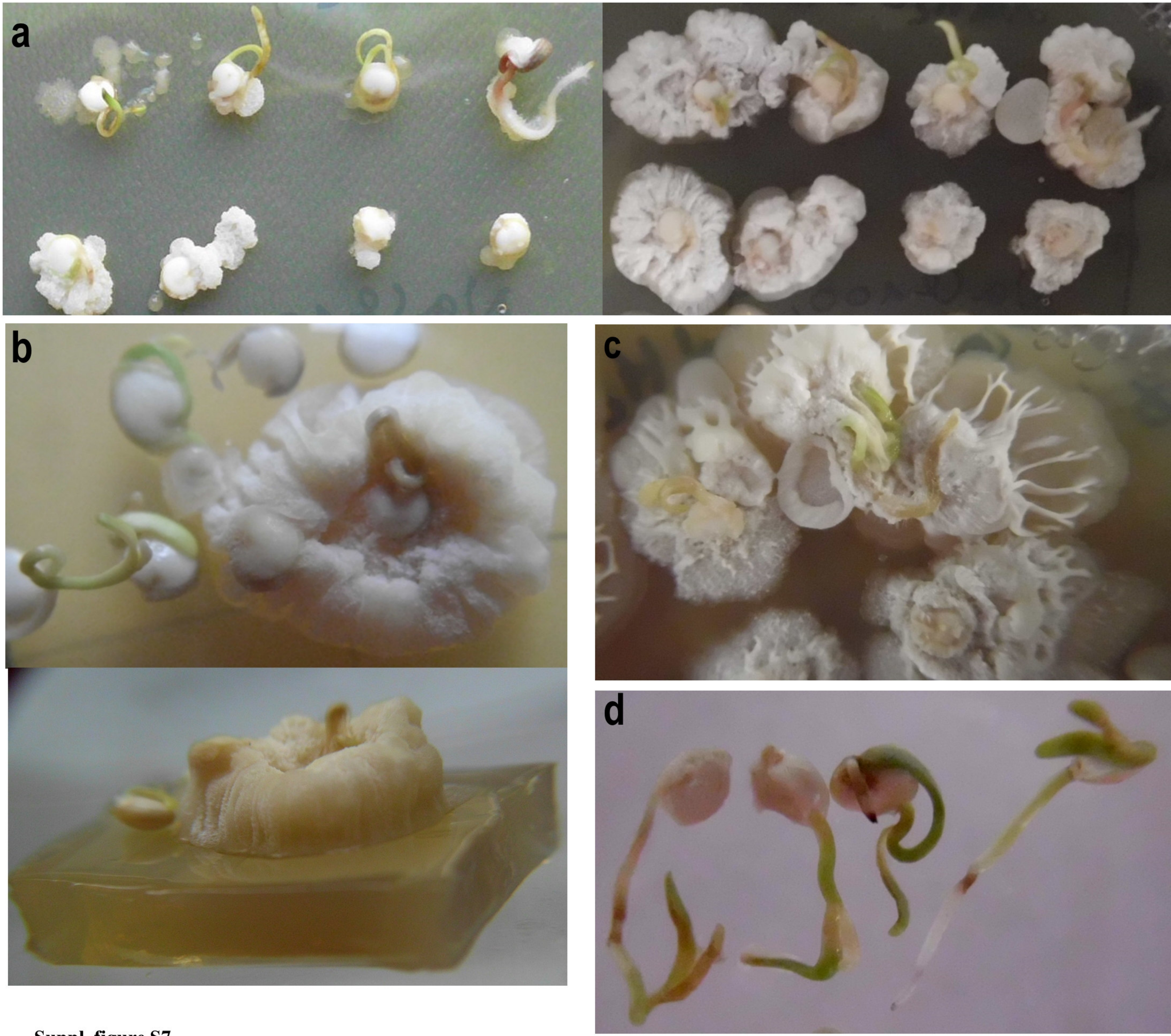

Suppl. figure S7

Growth of quinoa on YPD and progressive colonization by seed-borne endophytes.

a) Seeds were imbibed in water for 24, transferred to YPD agar and photographed after 24h and 48h.

b) While drily-sown seeds (left) remain non-colonized, microorganisms rapidly proliferate on pre-imbibed seeds to form volcano-like structures (top- and side-view).

c) Seed(ling)s covered under massively proliferating colonies turn soft.

d) Moderately colonized seeds (from YPD agar, microbes wiped off prior to photography) whose leaves had reached into the air retained a rigid tissue texture.

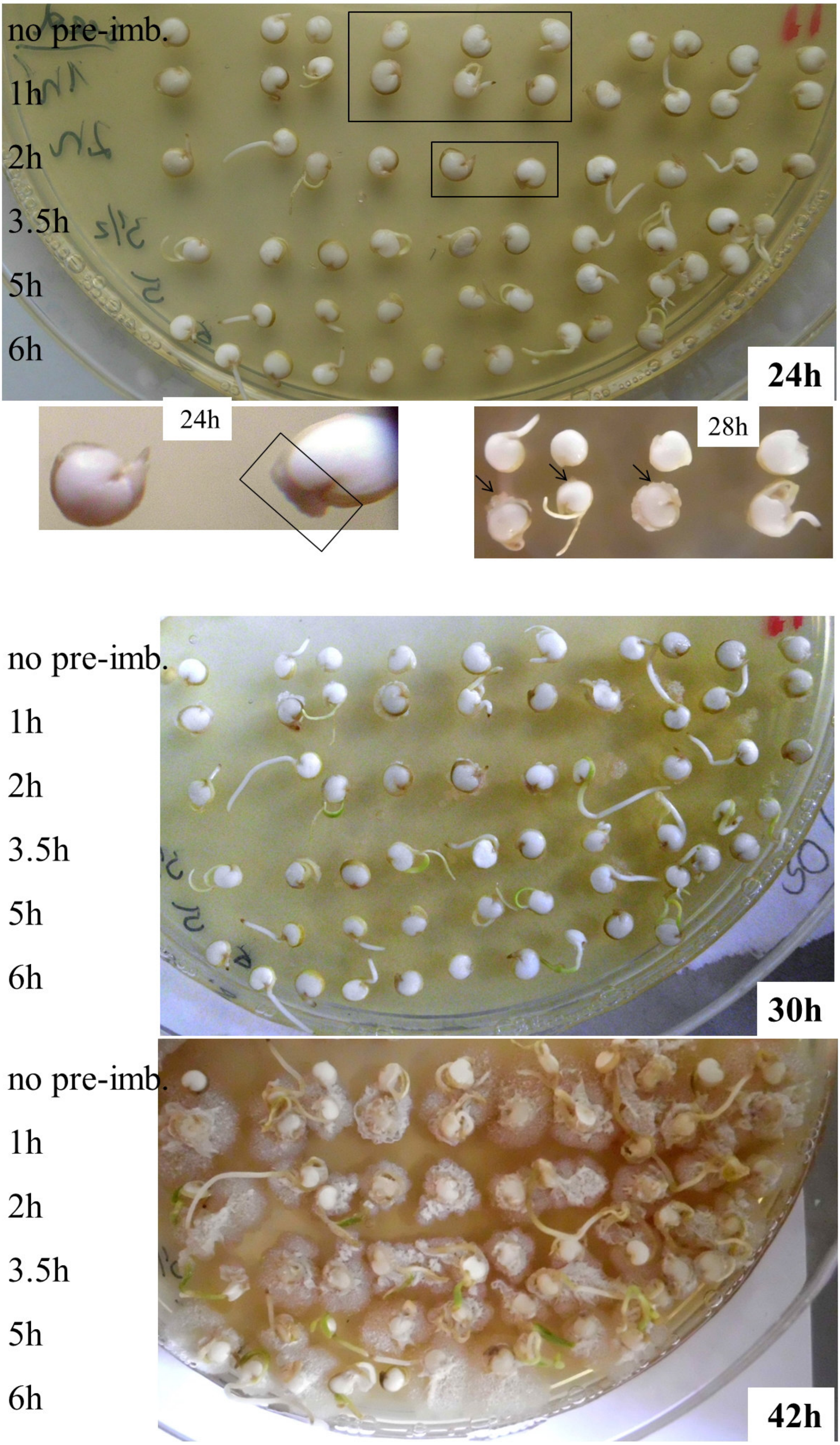

Suppl. Figure S8

Pre-imbibition favours proliferation of seed-borne microbes.

Quinoa seeds were placed on YPD agar, directly or after imbibition in water for 1 to 6 hours. The plate was photographed after 24h, 30h and 42h. Magnification/arrow: first colonies detectable at the seed surface of one seed after 24h, on several seeds after 28h.
